# Updated checklist of polychaete species (Annelida) recorded from Malaysia, with remarks on the research history

**DOI:** 10.3897/BDJ.11.e110021

**Published:** 2023-10-19

**Authors:** Raz Shauqeena Batrisyea Razmi Shah, Yusof Shuaib Ibrahim, Tulio F. Villalobos-Guerrero, Masanori Sato

**Affiliations:** 1 Faculty of Science and Marine Environment, Universiti Malaysia Terengganu, 21030, Kuala Nerus, Kuala Terengganu, Malaysia Faculty of Science and Marine Environment, Universiti Malaysia Terengganu, 21030, Kuala Nerus Kuala Terengganu Malaysia; 2 Department of Marine Ecology, Centro de Investigación Científica y de Educación Superior de Ensenada, 22860, Ensenada, Baja California, Mexico Department of Marine Ecology, Centro de Investigación Científica y de Educación Superior de Ensenada, 22860 Ensenada, Baja California Mexico; 3 Department of Earth and Environmental Sciences, Graduate School of Engineering and Science, Kagoshima University, 1-21-35 Korimoto, 890-0065, Kagoshima, Japan Department of Earth and Environmental Sciences, Graduate School of Engineering and Science, Kagoshima University, 1-21-35 Korimoto, 890-0065 Kagoshima Japan

**Keywords:** bathymetric, bristleworm, compilation, freshwater, inventory, marine, north Borneo, new records, Strait of Malacca

## Abstract

**Background:**

An updated comprehensive checklist of polychaete species, which have been recorded from Malaysian waters, is provided, with their geographic distributions and the research history for them. A total of 57 species belonging to 30 families have been reported since the early 1870s, with Nereididae as the most dominant family with ten species; however, more than half of the total are questionable species in the country. Despite the increased efforts of polychaete studies in the past decade, the taxonomic endeavour of discovering and describing species in the country could be higher. Malaysian polychaetes were mostly recorded from Peninsular Malaysia, whereas very few were from Borneo Island. Most previously recorded species were associated with intertidal and estuarine habitats and a few were found in the subtidal and freshwater environments. We stress the need for urgent research on this biologically, ecologically and culturally relevant taxonomic group as the species accumulation curve grows exponentially in this megadiverse country.

**New information:**

The current checklist has been updated since the previous one in 2013. Many species previously listed were judged as doubtful and not taxonomically reliable.

## Introduction

Polychaetes are predominantly marine, with some species inhabiting fresh and terrestrial groundwaters (Glasby et al. 2021). This taxon is one of the most abundant and diverse groups in the benthic community of marine environments, with more than 12,000 valid species within over 80 families worldwide (Read and Fauchald 2023). They include sensitive and tolerant species that are useful as bioindicators to detect some changes in habitat environments (Reish 1984, Pocklington and Wells 1992). Some polychaetes have been economically utilised as bait worms and aquaculture feed for fish (Olive 1994, Olive 1999).

Malaysia is one of the world’s megadiverse countries (Convention on Biological Diversity (CBD) 2023). Despite being very well represented in the marine environment (Glasby et al. 2000), the records of polychaetes are quite unsatisfactory. This is due to the lack of efforts in former and current expeditions. Besides, very few researchers are interested in studying the taxonomy of Malaysian polychaete species.

The latest previous checklist of polychaetes in Malaysia was published by Idris and Arshad (2013), which included the records and distribution of species from the earliest in 1866 to the latest in 2013. The records were done by scientists from numerous countries and research collaborations. This pioneering study was relevant to understand the knowledge of polychaetes in the country. However, some records were overlooked and many were still considered cosmopolitan species, which was the paradigm until the late 1980s (see Salazar-Vallejo (2020), Salazar-Vallejo and González-Vallejo (2020)). The studies on Malaysian polychaetes have increased and the systematics of polychaetes have improved considerably in the past ten years. To generate a checklist of polychaete only for Malaysia is important for ecological management, biodiversity assessment and monitoring and other analyses pertinent to biodiversity. Therefore, a more comprehensive compilation checklist of polychaete species in Malaysian waters that analyses their current taxonomic status and distribution is still required.

Here, an updated checklist of Malaysian polychaetes is provided, re-evaluating the previous checklist and adding current information.

## Materials and methods

Previous taxonomic and ecological papers (until 2022), in which Malaysian polychaetes were identified to the species level by taxonomic specialists with or without descriptions of specific characteristics, were analysed. In the cases where records were tentatively treated at genus level in literature, only those with taxonomic remarks were here included (e.g. *Prionospio* sp. in Ong (1995)). This checklist comprises marine, estuarine and freshwater species reported in Malaysia. Species originally described outside the Central and Western Indo-Pacific biogeographic regions, realms to which Malaysian coastal waters belong, are regarded as questionable records until their present distribution in the country is re-assessed. These species are noted in Suppl. material 1. The information about each species described was confirmed with the original description and subsequent literature. Additionally, an indication of the specimens’ repository deposition used in the species original record was incorporated to the list (Table 4), when available. This paper divides Malaysia into two main geographic parts: Peninsular Malaysia (including the east and west coasts of Peninsular Malaysia) and Borneo (including Sabah and Sarawak).

A bathymetry distribution map of species was constructed using ArcGIS 10.8 and the bathymetry data provided by the General Bathymetric Chart of the Oceans (GEBCO). The bathymetric information of the polychaete was arranged by a 10 m depth interval. The distribution was shown by the following five habitat zones: estuary, intertidal, subtidal, freshwater and unknown zones (Suppl. material 2). The single freshwater species was not given to its exact locality and type habitat; however, it was still shown in a separate map since it has important significance in this checklist. The unknown zone category consisted of species whose habitat was not mentioned or unknown and their locality and depth were estimated, based on the location given by the original author. Most species were plotted to their specific depth and locality. Meanwhile, some species' depth, locality or both were estimated, based on location recorded. A accumulative curve of species recorded over time and the exponential curve line were plotted using Microsoft Excel.

**History of polychaete research in Malaysia**

The earliest studies of polychaetes in Malaysia began with scientists of different nationalities before the independence era (the 1950s). The first study focused on polychaetes at the Strait of Malacca, considered as one of the busiest shipping lanes in the world, following behind the Dover Strait back in those days (Freeman 2003, Ismail and Mohd Sani 2010). The eminent French naturalist Armand de Quatrefages, who worked in the Muséum National d’Histoire Naturelle, Paris (MNHN), described the first polychaete species from this region, *Iphionecimex* de Quatrefages, 1866 (= *Gaudichaudiuscimex* sensu Pettibone, 1986). The single specimen is currently deposited at the MNHN (IA-TYPE 0326), which was collected probably in Pulau Pinang (Malaysia) or Singapore on any date between 17 and 26 February 1836 by Charles Gaudichaud-Beaupré in a circumnavigation carried out on board the French corvette *La Bonite* (Eydoux and Souleyet 1842).

There were expeditions in the IMPA (Indo-Malay-Philippines Archipelago) or the Malay Archipelago (Wallace 1869). One of them was the Dutch “Siboga” (1899–1900) expedition that gathered 782 polychaete species from Indonesia, 269 species being considered new to science (see Bleeker and van der Spoel (1992), Glasby and Al Hakim (2017)). A few decades later, more international expeditions to the Malay Archipelago were carried out, such as the Dutch “Snellius” expedition (1929–1930), which visited the same area as “Siboga”, the U.S. Fisheries steamer “Albatross” in late 1907, collected most samples from the Gulf of Tomini (northern Sulawesi), the Philippines waters, the northern coast of Borneo and the Moluccas and the Danish “Dana” expedition sampled in Indo-Malay waters in 1929 (Dana-Report 1934). In the post-1960s, there were numerous collaborative research studies, particularly from adjacent countries (Thailand, Indonesia and the Philippines) with the United States, Australia and European countries (Glasby and Al Hakim 2017). However, records of polychaetes in Malaysian waters from these expeditions were insufficient.

Various national and international research studies were undertaken in the early 1960s up to recent years (Table 1). Most were carried out on the west coast of Peninsular Malaysia. Chuang (1961), Sasekumar (1974), Nakao et al. (1989a), Ong (1995), Nishi (1999), Rezai et al. (2002) and Glasby and Hsieh (2006) collected polychaetes along the coastlines of the Strait of Malacca. On the other hand, Nakao et al. (1989b) collected from the east coast of Peninsular Malaysia.

Almost a decade later, Idris and Arshad (2013) compiled the first polychaete checklist in Malaysia, based on literature records. They reported 64 species belonging to 31 families and included a re-description of two commercially exploited onuphid species: *Diopatraclaparedii* Grube, 1878 and *Hallaokudai* Imajima, 1967. Afterwards, two new species were described from the west coast of Peninsular Malaysia: the eunicid *Marphysamoribidii* Idris, Hutchings and Arshad, 2014 from the mangrove area of Morib (Idris et al. 2014) and the sabellarid *Sabellariajeramae* Nishi, Matsuo, Capa, Tomioka, Kajihara, Kupriyanova and Polgar, 2015 from the intertidal zone of Jeram (Nishi et al. 2015). Later on, new recorded species were done by Ibrahim et al. (2019) who registered the nereidid *Neanthesglandicincta* (Southern, 1921), originally described from Indian waters, in the eastern coast of Peninsular Malaysia, whose reproductive biology, epitokal morphology and swimming behaviour was later described in detail (Azmi et al. 2021). In addition, Idris et al. (2022) studied the serpulid *Spirobranchuscorniculatus* (Grube, 1862) from the east coast of Peninsular Malaysia.

The accumulative curve of the records of species in Malaysia (Fig. 2) indicates that studies on polychaetes attracted the attention of researchers during the 1960s. From there, the records started to increase gradually until 2019. In the past decade, a markedly drastic increase in studies of polychaetes on different topics (ecology, environment and diversity-related) was upscaled due to the efforts carried out by local scientists (e.g. Gholizadeh et al. (2012), Shi et al. (2014), Polgar et al. (2015), Hamzah et al. (2021)). Contrarily, there have been only a few studies addressing taxonomy throughout the years. Malaysia is the type locality for ten species, which were mostly described from the Peninsular Malaysia (de Quatrefages 1866, Grube 1875, Horst 1911, Rullier 1969, Rullier 1970, Jones 1974, Rullier 1976, Nishi 2001, Idris et al. 2014, Nishi et al. 2015). After the 1950s, researchers such as Sasekumar (1974), Nakao et al. (1989a), Nakao et al. (1989b) and Ong (1995) contributed to increasing the number of Malaysian species with various literature.

## Checklists

### Checklist of Polychaeta from Malaysia

#### Amphinomidae

19D78CB1-45D5-5A1A-9B8E-B692E8CD2DB6

#### Notopygos cirratus

Horst, 1911

ED3FDC14-C725-54AE-BD52-0749F99CEDEA

##### Distribution

Type locality. North Ubian Islands, Malaysia.

Distribution in Malaysia. North Ubian Islands, Malaysia.

Distribution outside Malaysia. Only from type locality (Horst 1911, Salazar-Vallejo et al. 2014).

#### Aphroditidae

0887BF81-CAC1-5157-BDE2-73E129B69BD9

#### Aphrodita sondaica

Grube, 1875

4A70F460-27D8-544F-9976-5DCBB6C915E5

##### Distribution

Type locality. North Borneo, Malaysia; Palawan, Philippines.

Distribution in Malaysia. North Borneo, Malaysia (Grube 1875).

Distribution outside Malaysia. Philippines (Grube 1875); Indonesia (Pamungkas and Glasby 2019).

#### Capitellidae

E45E24A6-CA0D-5C2B-A5A3-314D0D8CFBD0

#### Notomastus latericeus

Sars, 1851

603EA6FA-E3E0-5D74-9FA1-DB133BAD924D

##### Distribution

Type locality. Komagfjord, Norway.

Distribution in Malaysia. Questionable record: Kuala Terengganu River estuary, Terengganu (Nakao et al. 1989b, Idris and Arshad 2013).

Distribution outside Malaysia. Italy (Giangrande and Fraschetti 1993); England (Pienkowski 1983); Turkey (Çinar et al. 1998); Around India (Fauvel 1953); China (Yang and Sun 1988); Japan (Imajima and Hartman 1964).

#### Chaetopteridae

E1A4FC16-BEB2-5B4E-8110-733F66F44404

#### Mesochaetopterus selangora

(Rullier, 1976)

92675A11-9B3E-5130-A828-59F0F87B2993

##### Distribution

Type locality. Morib, Selangor, Malaysia.

Distribution in Malaysia. Only from type locality (Rullier 1976, Nishi 1999, Idris and Arshad 2013).

##### Notes

Endemic species. Previously recorded as *Sasekumariaselangora* Rullier, 1976. The porcellanid crab *Polyonyxvermicola* Ng & Sasekumar, 1993 is an obligate commensal of tubes of the chaetopterid worm (Ng and Sasekumar 1993).

#### Spiochaetopterus costarum

(Claparède, 1869)

78313E53-6891-5622-B871-539D2262D883

##### Distribution

Type locality. Mediterranean Sea, Italy.

Distribution in Malaysia. Questionable record: Selangor River estuary (Nakao et al. 1989a).

Distribution outside Malaysia. Japan (Nishi and Arai 1996).

#### Cirratulidae

E4B1ADCF-28A3-5474-8DAC-522E1C08109B

#### Cirriformia tentaculata

(Montagu, 1808)

2EF98D57-8F47-5887-BB2D-A28E77ABB3E4

##### Distribution

Type locality. South coast of Devonshire, United Kingdom.

Distribution in Malaysia. Questionable record: Kuala Terengganu River estuary, Terengganu (Nakao et al. 1989b, Idris and Arshad 2013).

Distribution outside Malaysia. China (Yang and Sun 1988); Thailand (Angsupanich and Kuwabara 1999); India (Murugesan and Balasubramanian 2021); Canary Islands (Esquete et al. 2016); Western coast of Tunis Bay (Afli et al. 2013).

#### Dorvilleidae

1E8BC451-5006-580A-9713-9DCE6E89A6CD

#### Protodorvillea egena

(Ehlers, 1913)

BF37B4ED-ACCA-5D60-BA8B-E50E65093E83

##### Distribution

Type locality. Simonstown, South Africa.

Distribution in Malaysia. Questionable record: Teluk Aling, Penang (Ong 1995, Idris and Arshad 2013).

Distribution outside Malaysia. Australia (Lewis et al. 1981); India (Murugesan and Balasubramanian 2021).

#### Eulepethidae

6CB98700-C816-5B7A-90D6-99056A7AC2D9

#### Grubeulepis malayensis

Nishi, 2001

FF47FFD3-CACB-5D6A-A3C1-88FA1A031B16

##### Distribution

Type locality. Morib Beach, Selangor, Malaysia.

Distribution in Malaysia. Only from type locality (Nishi 2001, Idris and Arshad 2013).

##### Notes

Living in chaetopterid tubes.

#### Eunicidae

46C7187B-C4F1-5CDD-8BA4-71349BAED3FA

#### Marphysa cf. mossambica

(Peters, 1854)

967F6745-A6CE-5B46-BC0F-8A8DF5CBDD13

##### Distribution

Distribution in Malaysia. Along west of Peninsular Malaysia (Idris et al. 2014).

#### Marphysa moribidii

Idris, Hutchings & Arshad, 2014

EDE375D0-85A4-59BF-BE6D-D21359A273E0

##### Distribution

Type locality. Morib, Selangor, Malaysia.

Distribution in Malaysia. Morib, Selangor and Pengkalan Balak, Malacca (Idris et al. 2014).

##### Notes

Locally known as ‘Ruat bakau’ and harvested as bait worms. Previously identified as Marphysacf.sanguinea by Idris et al. (2014).

#### Glyceridae

5C325372-7ED0-5E37-A374-FE6A060F5772

#### Glycera alba

(O.F. Müller, 1776)

DD03B828-E90E-550F-BAC5-3618421C65D7

##### Distribution

Type locality. Norway (no precise locality).

Distribution in Malaysia. Questionable record: Selangor River estuary; Kuala Terengganu River estuary, Terengganu (Nakao et al. 1989a, Nakao et al. 1989b, Böggemann 2002, Idris and Arshad 2013).

Distribution outside Malaysia. Mozambique (Macnae and Kalk 1962); Vietnam (Phan 2015); Scotland (Blackstock and Barnes 1982); India (Harkantra and Parulekar 1985).

#### Glycera cinnamomea

Grube, 1874

690BD211-9213-5445-83F8-A43D08053533

##### Distribution

Type locality. Indian Ocean, Sri Lanka.

Distribution in Malaysia. Teluk Aling, Penang (Ong 1995, Idris and Arshad 2013).

Distribution outside Malaysia. Andaman, Nicobar coast (Böggemann and Eibye-Jacobsen 2002); Myanmar (Böggemann 2002); Arabian Gulf (Wehe and Fiege 2002); Indian Ocean, Red Sea, Persian Gulf, East and South China Sea (Böggemann and Eibye-Jacobsen 2002); Australia (Neave et al. 2013).

##### Notes

Previously recorded as *Glyceraprashadi* Fauvel, 1932.

#### Iospilidae

CDEBD018-3529-51ED-9472-E7F59E6CD621

#### Phalacrophorus uniformis

Reibisch, 1895

F540E15C-7837-5B70-96BF-EA9671E58A69

##### Distribution

Type locality. Tropical Atlantic.

Distribution in Malaysia. Questionable record: Strait of Malacca (Dales 1959, Rezai et al. 2002, Idris and Arshad 2013).

Distribution outside Malaysia. Mexico (Fernández-Álamo and Sanvicente-Añorve 2005); Eastern Brazlilian coast (Tovar-Faro et al. 2013).

#### Lopadorrhynchidae

3B7980A0-FCB3-508D-A703-04CA84949AC2

#### Lopadorrhynchus brevis

Grube, 1855

60AEF568-6204-55C9-A470-C0000D43FDE1

##### Distribution

Type locality. Mediterranean Sea.

Distribution in Malaysia. Questionable record: Strait of Malacca (Dales 1959, Rezai et al. 2002, Wehe and Fiege 2002, Idris and Arshad 2013).

Distribution outside Malaysia. Red Sea, Gulf of Aden (Wehe and Fiege 2002); Mexican Pacific (Hernández-Alcántara et al. 2008); Gulf of California (Fernández-Álamo 1991).

#### Maupasia coeca

Viguier, 1886

5C78C287-8961-5720-ABB6-BF9EF974B24A

##### Distribution

Type locality. Algiers Bay, Algeria.

Distribution in Malaysia. Questionable record: Strait of Malacca (Dales 1959, Rezai et al. 2002, Idris and Arshad 2013).

Distribution outside Malaysia. Western North Pacific (Amei et al. 2020); Off Northeast Brazil (Neumann-Leitão et al. 2008).

#### Pelagobia longicirrata

Greeff, 1879

4B9E296C-F964-56F4-8024-F57D50DE932E

##### Distribution

Type locality. Canary Islands, Tropical Atlantic.

Distribution in Malaysia. Questionable record: Strait of Malacca; Selangor River estuary (Dales 1959, Nakao et al. 1989a, Rezai et al. 2002, Idris and Arshad 2013).

Distribution outside Malaysia. Gulf of Aden, Arabian Sea (Wehe and Fiege 2002); Vietnam (Kolbasova and Neretina 2021); Atlantic Ocean (Pleijel and Dales 1991); Mediterranean Sea (Fauvel 1923, Pinca and Dallot 1995); South Adriatic (Batistić et al. 2004); Southern Chile (Bilbao et al. 2008); Mexico (Fernández-Álamo and Sanvicente-Añorve 2005); Scotia Front region (Siciński 1988); Strait of Magellan (Guglielmo et al. 2014).

#### Lumbrineridae

2ABE0EF1-5869-5BDE-94B1-5848AEB68C34

#### Gesaneris malaysiae

(Rullier, 1969)

36C4AAFF-5BB9-5550-BE45-BB4E71E7F43E

##### Distribution

Type locality. Port Swettenham (currently known as Port Klang), Selangor, Malaysia.

Distribution in Malaysia. Kapar mangrove forest, Klang, Selangor; Kuala Lumpur (Rullier 1969, Carrera-Parra 2006, Idris and Arshad 2013).

##### Notes

Previously recorded as *Lumbriconereismalayensis* (sic) Rullier, 1969 (Carrera-Parra 2006).

#### Lumbrinerides acuta

(Verrill, 1875)

2E41692E-C4D6-5645-B5D1-81D6D2E32C18

##### Distribution

Type locality. Rhode Island, United States.

Distribution in Malaysia. Questionable record: Kuala Terengganu River estuary, Terengganu (Nakao et al. 1989b, Idris and Arshad 2013).

Distribution outside Malaysia. Delaware Bay, United States (Kinner and Maurer 1978).

##### Notes

Previously recorded as *Lumbrinerisacuta* Verrill, 1875.

#### Nereididae

A6A357A8-D6B8-5E2F-BD3B-4C6940F96423

#### Dendronereides arborifera

Peters, 1854

DC598FCC-D4CB-54A8-AE4F-85D4C997AFE6

##### Distribution

Type locality. Mozambique, Indian Ocean.

Distribution in Malaysia. Selangor River estuary (Nakao et al. 1989a).

Distribution outside Malaysia. Singapore (Chan 2009); Africa (Pillay and Perissinotto 2008); Vasishta Godavari Estuary (Sarma and Rao 1982); India (Roy and Nandi 2012).

#### Namalycastis cf. abiuma

710E402A-AF03-50B0-B52D-AFC7C235BA0C

##### Distribution

Distribution in Malaysia. Pekan, Pahang (Idris et al. 2012, Idris and Arshad 2013).

#### Namalycastis rhodochorde

Glasby, Miura, Nishi & Junardi, 2007

4F38FD5E-2578-58D2-882A-D8697941FD51

##### Distribution

Type locality. Kalimantan, Indonesia.

Distribution in Malaysia. West coast of Peninsular Malaysia and Kota Kinabalu, Sabah, Borneo (Idris et al. 2012, Idris and Arshad 2013).

Distribution outside Malaysia. West Kalimantan, Indonesia (Junardi 2020).

##### Notes

Known as nypa palm worm and locally known as ‘Ruat nipah’ and ‘Punpun nipah’. Used as bait for fish and shrimp (Junardi et al. 2014).

#### Neanthes glandicincta

(Southern, 1921)

6B08562B-78B6-5B80-AE96-289C147B16BE

##### Distribution

Type locality. Near Calcutta, India.

Distribution in Malaysia. Jeram Beach, Selangor; Tumpat, Kelantan Delta, Kelantan; Setiu Lagoon, Terengganu; Kuala Ibai, Terengganu (Polgar et al. 2015, Ibrahim et al. 2019, Azmi et al. 2021).

Distribution outside Malaysia. India (Southern 1921, Lee and Glasby 2015); Myanmar (Monro 1937, Lee and Glasby 2015); Western Singapore (Lee and Glasby 2015); Thailand (Azmi et al. 2021).

##### Notes

Previously recorded as Ceratonereis (Composetia) burmensis Monro, 1937 (Polgar et al. 2015).

#### Perinereis aibuhitensis

(Grube, 1878)

89B048CF-9E5C-5D7A-AE96-49E968648D89

##### Distribution

Type locality. Aibuhit, Philippines.

Distribution in Malaysia. Setiu Wetlands, Terengganu (Ibrahim et al. 2017).

Distribution outside Malaysia. Taiwan, Philippines, Thailand, Indonesia, Australia (Grube 1878, Horst 1924, Russell 1962, Wu 1967, Hylleberg et al. 1986, Hutchings et al. 1991).

#### Perinereis cf. nuntia

(Lamarck, 1818)

8CE39C24-5687-53D6-B308-F7EE1D866714

##### Distribution

Distribution in Malaysia. Batu 4, Port Dickson, Negeri Sembilan (Idris et al. 2012, Idris and Arshad 2013).

##### Notes

Locally known as ‘Ruat pasir’ and ‘Punpun pasir’.

#### Perinereis cultrifera

(Grube, 1840)

834B1251-CF27-5A29-BD89-97B4578516A6

##### Distribution

Type locality. Gulf of Naples, Italy.

Distribution in Malaysia. Questionable record: Pulau Aur, Johor (Chuang 1961, Idris and Arshad 2013).

Distribution outside Malaysia. Algerian Mediterranean coast (Rouabah and Scaps 2003); Italy (Maltagliati et al. 2001); India (Elayaraja et al. 2010); France (Baert and Slomianny 1987); Morocco (Rouhi et al. 2008).

#### Perinereis rhombodonta

Wu, Sun & Yang, 1981

A4278E0D-86E3-5A2F-A084-33B488275570

##### Distribution

Type locality. Aotou, Guangdong; Beihai, Beilongwei and Qisha, GuangXi, China.

Distribution in Malaysia. Blue Lagoon, Port Dickson, Negeri Sembilan (Glasby and Hsieh 2006, Idris and Arshad 2013).

Distribution outside Malaysia. China (Wu et al. 1981); Hong Kong, Thailand, Singapore, Indonesia (Glasby and Hsieh 2006).

#### Platynereis bicanaliculata

(Baird, 1863)

400315C0-CB6C-525B-8008-8B5CF78C41D8

##### Distribution

Type locality. Vancouver, Canada.

Distribution in Malaysia. Questionable record: Kuala Terengganu River estuary, Terengganu (Nakao et al. 1989b, Idris and Arshad 2013).

Distribution outside Malaysia. United States (Roe 1975, Fong 1993); Japan (Fukao 1996).

#### Pseudonereis variegata

(Grube & Kröyer in Grube, 1857)

BFC445E9-4C67-586D-A241-C2756DECF3E0

##### Distribution

Type locality. Valparaíso, Chile.

Distribution in Malaysia. Questionable record: Pulau Aur, Johor (Chuang 1961, Idris and Arshad 2013).

Distribution outside Malaysia. Chile (Grube 1857); southern Africa (Herwerden 1989); Egypt (Abdelnaby 2020b); Pakistan (Siddiqui and Mustaquim 1988).

#### Oenonidae

4DADA735-FC01-5781-BC7B-23E2C7ECCAEB

#### Halla okudai

Imajima, 1967

3275D0EC-3607-51CE-B52C-1F51328EB73A

##### Distribution

Type locality. Seto Inland Sea, Japan.

Distribution in Malaysia. Questionable record: Malacca (Okuda 1933, Idris and Arshad 2013).

Distribution outside Malaysia. Japan (Okuda 1933, Kobayashi et al. 2020); China (Saito et al. 2014); southern Australia (Paxton 2009).

##### Notes

Locally known as ‘Ruat beting’ in Malacca, Malaysia.

#### Onuphidae

E865B185-E3D7-5513-B332-37516D45BBDB

#### Diopatra claparedii

Grube, 1878

744F5F88-0F88-5D93-B072-390E4D0E3E9C

##### Distribution

Type locality. Sungei Buloh, Singapore.

Distribution in Malaysia. Along mud-flats on the west coast of Peninsular Malaysia; Jeram Beach, Selangor (Paxton 2002, Idris and Arshad 2013, Polgar et al. 2015).

Distribution outside Malaysia. Singapore (Grube 1878); India (Pati et al. 2015).

##### Notes

Locally known as ‘Ruat sarung’.

#### Diopatra neapolitana

delle Chiaje, 1841

890C241F-35F0-58EC-982B-2E8C1CB78887

##### Distribution

Type locality. Gulf of Naples, Italy.

Distribution in Malaysia. Questionable record: Kapar mangrove forest, Klang; Teluk Aling, Penang (Sasekumar 1974, Ong 1995, Idris and Arshad 2013, Paxton and Arias 2017).

Distribution outside Malaysia. Red Sea, Indian Ocean (Wehe and Fiege 2002); Mediterranean Sea (Gambi and Giangrande 1986, Arvanitidis 2000, Dağli et al. 2005).

#### Orbiniidae

A44B0B2D-75E3-5294-B9AA-54982B2A4CBE

#### Leodamas chevalieri

(Fauvel, 1902)

94CD2F85-3397-5C99-839C-A4EEB3F8FED0

##### Distribution

Type locality. Casamance, Senegal.

Distribution in Malaysia. Questionable record: Teluk Aling, Penang (Ong 1995, Idris and Arshad 2013).

Distribution outside Malaysia. Western Africa (Fauvel 1902); Gulf of California (Hernández-Alcántara and Solís-Weiss 2013).

#### Naineris kalkudaensis

(De Silva, 1965)

BB3AF16D-48F8-59B0-80F3-62679AAD1A88

##### Distribution

Type locality. Kalkudah, Sri Lanka.

Distribution in Malaysia. Teluk Aling, Penang (Ong 1995, Idris and Arshad 2013).

Distribution outside Malaysia: Sri Lanka (De Silva 1965).

#### Phyllodocidae

FFC3D233-89E3-531F-BFB2-DA95E454D4B3

#### Plotohelmis capitata

(Greeff, 1876)

CA066FD1-C01F-596B-823F-1E68C5296D77

##### Distribution

Type locality. Algeria, Mediterranean Sea.

Distribution in Malaysia. Questionable record: Strait of Malacca (Dales 1959, Rezai et al. 2002, Idris and Arshad 2013).

Distribution outside Malaysia. Mediterranean, warm North Atlantic, Japan (Day 1967).

#### Rhynchonereella moebii

(Apstein, 1893)

B96E3387-9C54-5ED0-90E1-87C50141E4F2

##### Distribution

Type locality. Sicily, Italy.

Distribution in Malaysia. Questionable record: Strait of Malacca (Dales 1959, Rezai et al. 2002, Idris and Arshad 2013).

Distribution outside Malaysia. Mediterranean Sea, tropical and subtropical Atlantic and Pacific Oceans, India (Jiménez-Cueto and Suárez-Morales 2008).

#### Vanadis minuta

Treadwell, 1906

AF34C67A-36D9-594C-84EB-982600FE14C2

##### Distribution

Type locality. Mediterranean Sea.

Distribution in Malaysia. Questionable record: Strait of Malacca (Dales 1959, Rezai et al. 2002, Idris and Arshad 2013).

Distribution outside Malaysia. Pacific and Atlantic Oceans (Dales 1957b); Bangka Straits, East of Sumatra, West of Borneo, Singapore, Natuna Islands, South China (including Hong Kong, southern Taiwan, Paracel Islands) (Glasby et al. 2016).

#### Pilargidae

889C1A96-A956-5E59-9324-1835AD22FCEC

#### Sigambra ocellata

(Hartmann-Schröder, 1959)

84C1059E-A735-51B8-A6B1-96841E986522

##### Distribution

Type locality. El Salvador, Pacific Ocean.

Distribution in Malaysia. Questionable record: Selangor River estuary (Nakao et al. 1989a).

Distribution outside Malaysia. Central America (Pettibone 1966); El Salvador (Rivera and Rivera 2008).

#### Poecilochaetus serpens

Allen, 1904

BE4B490B-B142-5083-822E-DB7EC62EC31B

##### Distribution

Type locality. Plymouth, England.

Distribution in Malaysia. Questionable record: Teluk Aling, Penang (Ong 1995, Idris and Arshad 2013).

Distribution outside Malaysia. English Channel, Irish Sea, Skagerrak Azores, Canary Island, Mediterranean Sea, South Africa, Gulf of Mannar, Waltair (Achari 1968); Arabian Gulf (Mohammad 1980); Egypt (Abdelnaby 2020a).

#### Poecilochaetidae

F75C777A-3B05-5843-8F77-CF5F10E2EE3E

#### Polynoidae

493B6A72-4CC3-583A-BEA1-AB2A388B49CF

#### Drieschia pelagica

Michaelsen, 1892

1BDD7904-97FA-52BD-9C3A-78713741DAB9

##### Distribution

Type locality. Sri Lanka, Indian Ocean.

Distribution in Malaysia. Strait of Malacca (Dales 1959, Rezai et al. 2002, Idris and Arshad 2013).

Distribution outside Malaysia. New England (Pettibone 1963); Marmara, Aegean Seas (Wesenberg-Lund 1939, Çinar et al. 2014).

#### Gaudichaudius cimex

(de Quatrefages, 1866)

7E752D2E-B719-514B-88DC-EE45CFBC7139

##### Distribution

Type locality. Strait of Malacca, Malaysia.

Distribution in Malaysia. Only from type locality (Pettibone 1986, Solis-Weiss et al. 2004, Idris and Arshad 2013, Salazar-Vallejo et al. 2014).

Distribution outside Malaysia. Digha coast (Sarkar and Talukdar 2003); Indo-West Pacific (Pettibone 1986, Martin and Britayev 1998); Yellow Sea, South China Sea, Vietnam (Fauvel 1932, Uschakov and Wu 1959, Uschakov and Wu 1965, Uschakov and Wu 1979, Yang and Sun 1988); Hainan Island (Barnich et al. 2004).

##### Notes

Originally recorded as *Iphionecimex* de Quatrefages, 1866.

#### Olgalepidonotus kumari

(Rullier, 1970)

4B02827D-7D87-588A-BCBF-112EB0FA96C3

##### Distribution

Type locality. Port Swettenham (currently known as Port Klang), Selangor, Malaysia.

Distribution in Malaysia. Kapar mangrove forest, Klang; Port Klang, Selangor (Rullier 1970, Sasekumar 1974, Pettibone 1995, Idris and Arshad 2013).

Distribution outside Malaysia. Thailand (Sasekumar 1974).

##### Notes

Previously recorded as *Lepidonotuskumari* Rullier, 1970.

#### Sabellariidae

381D61B1-9AA2-571C-A053-595738BD94CC

#### Sabellaria jeramae

Nishi, Matsuo, Capa, Tomioka, Kajihara, Kupriyanova & Polgar, 2015

1FF4F53B-29F0-5AD8-86C0-A12390CC82EF

##### Distribution

Type locality. Jeram Beach, Selangor, Malaysia.

Distribution in Malaysia. Only from type locality (Nishi et al. 2015).

#### Sabellidae

9334AEC0-1DD0-591C-91D5-5EB9E07883EC

#### Branchiomma nigromaculatum

(Baird, 1865)

30F2F4EC-195B-50FD-9C38-4F5E3EF62DDE

##### Distribution

Type locality. Saint Vincent and the Grenadines, Caribbean Sea.

Distribution in Malaysia. Questionable record: Teluk Aling, Penang (Ong 1995, Idris and Arshad 2013).

Distribution outside Malaysia. Colombian Caribbean (Londoño-Mesa et al. 2002).

#### Caobangia abbotti

Jones, 1974

7A271182-50A5-57C2-8C57-F3DD8EAEE10F

##### Distribution

Type locality. Ranau, Sabah, Malaysia.

Distribution in Malaysia. Kinabatangan and Gunung Kinabalu, Sabah and Sarawak (Jones 1974, Idris and Arshad 2013).

Distribution outside Malaysia. Philippines (Rouse 2004).

##### Notes

Currently, the only freshwater species in Malaysia. Paratypes collected from the Robin River at Dana Amu in Sarawak (Jones 1974).

#### Scalibregmatidae

8BA503C8-EBF0-51A6-9A4E-D6C99BD731A2

#### Parasclerocheilus branchiatus

Fauvel, 1928

9D71E128-9610-57CE-BFB7-FB1BBDF62042

##### Distribution

Type locality. Shingle Island, India.

Distribution in Malaysia. Teluk Aling, Penang (Ong 1995, Idris and Arshad 2013).

Distribution outside Malaysia. Gulf of Oman (Fauvel 1932, Cantone 1982); Israel, India, Somalia (Cantone 1982).

#### Sigalionidae

293959E4-7CDD-567F-A49B-465F0E6DD300

#### Euthalenessa digitata

(McIntosh, 1885)

E0616A57-3EEC-5774-B435-D4075FA4AF73

##### Distribution

Type locality. Admirality Islands, Bismarck Archipelago, Papua-New Guinea.

Distribution in Malaysia. Teluk Aling, Penang (Ong 1995, Wehe and Fiege 2002, Idris and Arshad 2013).

Distribution outside Malaysia. Western Pacific Ocean, Red Sea, Persian Gulf, Gulf of Oman, Andaman Sea, Gulf of Thailand, Japan (Aungtonya et al. 2010).

#### Pisione oerstedii

Grube, 1857

FA5B00F6-860C-5FCC-8F9F-E408920C8E69

##### Distribution

Type locality. Valparaíso, Chile.

Distribution in Malaysia. Questionable record: Teluk Aling, Penang (Ong 1995, Idris and Arshad 2013).

Distribution outside Malaysia. Peru, Chile, New Zealand, India, South China Sea (Wu et al. 1998).

#### Spionidae

F8FE65DD-D97B-5764-8397-77A90B8FCD1D

#### Paraprionospio pinnata

(Ehlers, 1901)

E205815E-ACB0-542F-8A64-A5AE7FADDBF3

##### Distribution

Type locality. Talcahuano, Chile.

Distribution in Malaysia. Questionable record: Selangor River estuary; Kuala Terengganu River estuary (Nakao et al. 1989a, Nakao et al. 1989b, Idris and Arshad 2013).

Distribution outside Malaysia. Korea (Lim and Hong 1997); Gulf of Mexico (Baustian et al. 2018).

#### Prionospio sp.

381C2997-2270-5D67-8A83-10E3320BC8D9

##### Distribution

Distribution in Malaysia. Teluk Aling, Penang (Ong 1995, Idris and Arshad 2013).

##### Notes

It showed similar features to *P.malmgreni* Claparède, 1869; however, the author, Ong (1995) needed clarification and samples were undetermined.

#### Sternaspidae

45003EDF-1EB3-5269-9A83-2ECEC2F3DF10

#### Sternaspis scutata

(Ranzani, 1817)

11A505C7-E29A-55DD-9527-E60DA6086988

##### Distribution

Type locality. Turkey, Izmar Bay, Aegean Sea.

Distribution in Malaysia. Questionable record: Selangor River estuary; Kuala Terengganu River estuary (Nakao et al. 1989a, Nakao et al. 1989b, Idris and Arshad 2013).

Distribution outside Malaysia. Mediterranean Sea to the English Channel (Townsend et al. 2006, Sendall and Salazar-Vallejo 2013); Korea (Lim and Hong 1997).

##### Notes

It was re-described by Sendall and Salazar-Vallejo (2013) using neotype material, who suggested that records from non-Mediterranean or north-eastern Atlantic localities might belong to other, probably undescribed species.

#### Syllidae

E1DDF356-1C89-5CF3-8A17-6C9C764C4AB3

#### Syllis cornuta

Rathke, 1843

BA02B1BB-1F18-53FD-9CF7-00721D6EC926

##### Distribution

Type locality. Norway

Distribution in Malaysia. Questionable record: Teluk Aling, Penang (Ong 1995, Idris and Arshad 2013).

Distribution outside Malaysia. Norway (Rathke 1843); Iberian coasts (San Martin and López 2000).

#### Terebellidae

7E147149-FBDD-518A-B0F1-2DC57CBC0329

#### Lanice socialis

(Willey, 1905)

F125E8AF-D608-52A0-AC3A-7AB31F3ADE5B

##### Distribution

Type locality. Galle, Sri Lanka.

Distribution in Malaysia. Teluk Aling, Penang (Ong 1995, Idris and Arshad 2013).

Distribution outside Malaysia. Benin (Srikrishnadhas et al. 1987); India (Soota et al. 1981).

#### Tomopteridae

A4B26846-957E-5B5E-8A75-D6E1F81E0E5E

#### Tomopteris (Johnstonella) aloysii sabaudiae

Rosa, 1908

1586C454-CAF4-509D-8769-6F1BE0BDC3E8

##### Distribution

Type locality. Off Oaxaca, Mexico, Pacific Ocean.

Distribution in Malaysia. Questionable record: Strait of Malacca (Dales 1959, Rezai et al. 2002, Idris and Arshad 2013).

Distribution outside Malaysia. Mexican Pacific (Hernández-Alcántara et al. 2008).

#### Tomopteris (Johnstonella) dunckeri

Rosa, 1908

F4E737C3-FE15-5B06-ACDF-0813AFA7AF25

##### Distribution

Type locality. Sri Lanka, Indian Ocean.

Distribution in Malaysia. Strait of Malacca (Dales 1959, Rezai et al. 2002, Idris and Arshad 2013).

Distribution outside Malaysia. Sri Lanka (Rosa 1908); Vietnam (Phan 2015).

#### Tomopteris mariana

Greeff, 1885

08F6C170-D3DB-5F51-BDF3-1CFBC1E4FE1E

##### Distribution

Type locality. Tropical Atlantic.

Distribution in Malaysia. Questionable record: Strait of Malacca (Dales 1959, Rezai et al. 2002, Idris and Arshad 2013).

Distribution outside Malaysia. China (Wu et al. 1980).

#### Tomopteris nisseni

Rosa, 1908

50D0CC60-F40A-58B7-8CBE-DAF2D18EF010

##### Distribution

Type locality. Off Brazil.

Distribution in Malaysia. Questionable record: Strait of Malacca (Dales 1959, Rezai et al. 2002, Idris and Arshad 2013).

Distribution outside Malaysia. California (Dales 1955); Spain (Dales 1957a).

#### Typhloscolecidae

81C8FC70-19E8-5938-AD22-DE900BFC2B89

#### Typhloscolex muelleri

Busch, 1851

7C83A470-E2A6-52D8-8D47-2F728D68FFCB

##### Distribution

Type locality. Trieste, Adriatic Sea.

Distribution in Malaysia. Questionable record: Strait of Malacca (Dales 1959, Rezai et al. 2002, Idris and Arshad 2013).

Distribution outside Malaysia. Atlantic Ocean (Orensanz and Ramírez 1973, Fernández-Álamo and Thuesen 1999); Southern Ocean (Rozbaczylo 1985); Pacific Ocean (Treadwell 1943, Dales 1957b, Tebble 1962).

## Analysis

### Polychaetes of Malaysian waters

A total of 57 species belonging to 47 genera in 30 families of polychaetes were recorded from Malaysian coastal waters; 53 species were recorded from Peninsular Malaysia, three species from Borneo and one species from both geographical regions. Amongst them, ten species (17% of total) were originally described from Malaysia, whereas the presence of other 30 (53%) species is questionable.

### Bathymetric distribution of polychaetes in Malaysian waters

**Figure 1.**
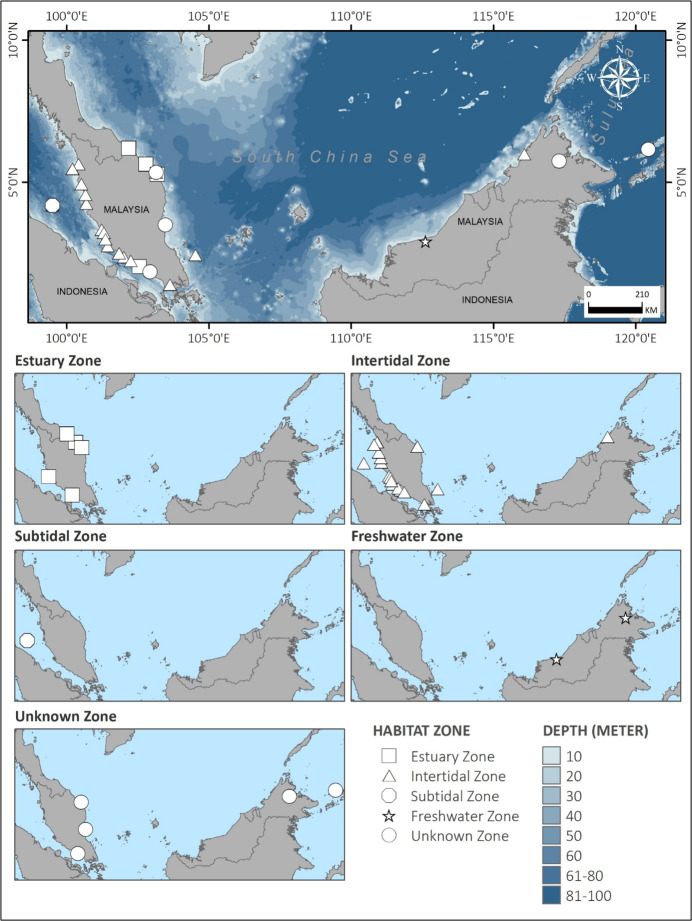
Bathymetric map distribution of Malaysian polychaetes on their type of habitat. Data provided by the General Bathymetric Chart of the Oceans (GEBCO).

The bathymetric information (Fig. 1) indicates that almost all species were recorded in less than 10 m depth, distributed at the estuarine and intertidal zones from the coastlines of Peninsular Malaysia. Species recorded at the subtidal zone were from the Strait of Malacca, with an estimated depth range of 30 m. The only freshwater species were recorded from Sabah and Sarawak. Notably, some species including those from Sabah, Sarawak and North Borneo, were not given their specific habitat type. Some species were collected from local bait shops. Other than that, *Caobangiaabboti*, *Marphysamoribidii*, *Neanthesglandicincta* and *Namalycastisrhodochorde* were recorded at more than one location in Malaysia.

### Species richness of polychaetes in Malaysian waters

The topmost species-rich polychaete family is Nereididae, with ten species (Table 2). Nereidids are amongst the most-rich species of polychaete families worldwide, alongside Syllidae, Polynoidae, Spionidae, Serpulidae and Terebellidae (Pamungkas et al. 2019), whereas, 16 families recorded one species only, all collected from Peninsular Malaysia: Amphinomidae, Aphroditidae, Capitellidae, Cirratulidae, Dorvilleidae, Eulepethidae, Iospilidae, Oenonidae, Pilargidae, Poecilochaetidae, Sabellariidae, Scalibregmatidae, Sternaspidae, Syllidae, Terebellidae and Typhloscolecidae.

## Discussion

Studies on marine annelids were mainly done by international scientists and through collaborations. Most local researchers focused on ecological and diversity studies. Over the past decades, few taxonomy studies were published particularly by Malaysian taxonomists (Ong 1995, Idris et al. 2012, Idris and Arshad 2013, Idris et al. 2014, Ibrahim et al. 2019). Although the number of recorded species has increased over the years, the number of described species is relatively low. Pamungkas and Glasby (2019) stated that Indonesia, Singapore and Malaysia have a common lack of funding by the government in direct taxonomic investigations unless linked to other economic or ecological focus research. This is understandable as marine taxonomy might not concern policy-makers (Pamungkas and Glasby 2019). To our concern, very few polychaete taxonomists and ecologists are currently active in Malaysia. As quoted by Buyck (1999), taxonomists are “endangered species”.

**Figure 2.**
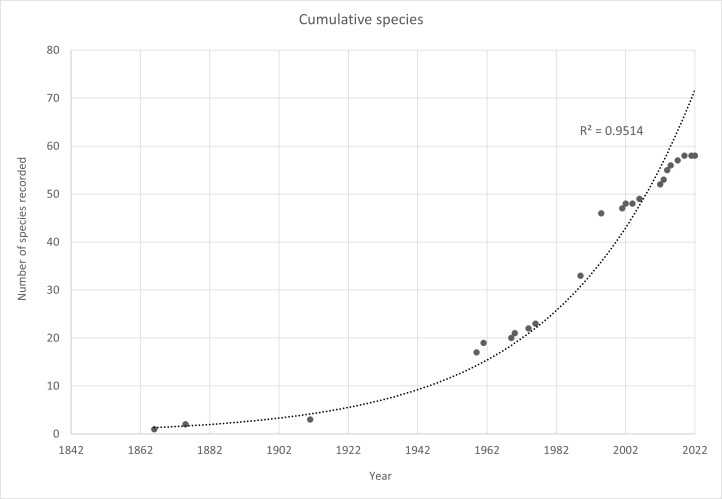
Cumulative number of species recorded over time in the Malaysian waters.

A total of 57 species of Malaysian polychaetes has been reported since 1866 until 2019. This current checklist included species from the previous one (Idris and Arshad 2013) excluding few species that were judged as doubtful. The species excluded were not provided with taxonomical description and species validation. After that, a few new species and records were reported (e.g. Idris et al. (2014), Nishi et al. (2015), Ibrahim et al. (2019)) and included in this current checklist. Comparing records in Malaysia with other countries, the former still falls behind on the polychaete species recorded amongst most of Southeast Asian countries (Table 3). The accumulation curve of polychaete species in Malaysia has not reached the asymptote, implying that more species are yet to be discovered (Fig. 2). The accumulative curve of the records of species in Malaysia and polychaete families reported less than ten species might also suggest that more research is needed to be done in both Peninsular Malaysia and Borneo Island.

More studies on polychaetes in Malaysia need to be carried out, mainly related to identifying and describing species. Additionally, there should be follow-up studies on several previous research to validate the species recorded in ecological and environmental studies. Rosli et al. (2018) recorded 340 polychaete species from the offshore of Pekan-Dungun, Kuala Terengganu and Kudat-Balambangan Island in both Peninsular Malaysia and Borneo, while Alias et al. (2022) reported 43 species in the Kuala Terengganu River estuary. However, their records consisted of unknown species. A further taxonomic investigation of polychaetes of these sites will increase the checklist considerably.

The presence of polychaete species considered to be widely distributed or cosmopolitan in Malaysian coastal waters has not been previously considered by this study. It is estimated that, of the total number of polychaete species recorded in Malaysia (57 species), approximately 53% (e.g. *Notomastuslatericeus*, *Spiochaetopteruscostarum*, *Cirriformiatentaculata*) require a detailed revision since some may belong to cryptic or pseudocryptic species (Knowlton 1993, Knowlton 2000), judging by their type locality. Global and regional revisions of polychaetes have revealed deficient taxonomy that caused the “cosmopolitan syndrome” (Williams 1984, Dauvin and Thiebaut 1994). Species from other distinct regions have been recorded in local waters using identification guides from distant areas, without carefully examining or comparing specimens or both. However, the detailed taxonomic revisions have aided in amending this syndrome and favoured the description of other local species. Unfortunately, as pointed out previously, Malaysia has been of limited interest to fellow polychaetologists throughout its natural history and the fact that more than half of the polychaete species recorded so far are questionable is a consequence of this gap in taxonomic studies.

International collaborations may help expand polychaete taxonomic research in the country. For instance, the involving of international and local polychaete taxonomists in identifying species and depositing biological materials in domestic and internationally recognised collections for future studies (Pamungkas and Glasby 2019). Besides, molecular taxonomy could also be applied in identifying species alongside morphological taxonomy. It has been demonstrated that the use of morphological and genomic techniques aids not only in understanding the polychaete biodiversity in particular regions, but also in disentangling the species hidden or obscured within cryptic species (Hutchings and Kupriyanova 2018, Simon et al. 2019). Moreover, to assess the effect of climate change, sea level rise and human-induced sea-level coastal zones, it is required to document species diversity on a regional basis (Al-Kandari et al. 2019).

In Malaysia, there is a need for more to be done in polychaete research. More studies on polychaete systematics, genetics, ecology, physiology and reproductive biology are urged to be undertaken. Acknowledging this taxonomic group as a vital member or the ‘keystone’ in the ecosystem is also essential. Their feeding behaviour, metabolic activities and distinct characteristics while modifying their habitats and surrounding made them known as the ecosystem engineer (Buchman et al. 2007, Fadhullah and Syakir 2016). Thus far, polychaetes have been applied in nanoscience technology and medicine (see Hussain et al. (2018), Pei et al. (2020), Che Soh et al. (2020)). By accentuating their importance and potential in various sectors, more research on polychaetes could be done in the future. This study is intended to aid in the assessment of marine biodiversity with a view to the protection and conservation of Malaysian waters.

## Supplementary Material

XML Treatment for Amphinomidae

XML Treatment for Notopygos cirratus

XML Treatment for Aphroditidae

XML Treatment for Aphrodita sondaica

XML Treatment for Capitellidae

XML Treatment for Notomastus latericeus

XML Treatment for Chaetopteridae

XML Treatment for Mesochaetopterus selangora

XML Treatment for Spiochaetopterus costarum

XML Treatment for Cirratulidae

XML Treatment for Cirriformia tentaculata

XML Treatment for Dorvilleidae

XML Treatment for Protodorvillea egena

XML Treatment for Eulepethidae

XML Treatment for Grubeulepis malayensis

XML Treatment for Eunicidae

XML Treatment for Marphysa cf. mossambica

XML Treatment for Marphysa moribidii

XML Treatment for Glyceridae

XML Treatment for Glycera alba

XML Treatment for Glycera cinnamomea

XML Treatment for Iospilidae

XML Treatment for Phalacrophorus uniformis

XML Treatment for Lopadorrhynchidae

XML Treatment for Lopadorrhynchus brevis

XML Treatment for Maupasia coeca

XML Treatment for Pelagobia longicirrata

XML Treatment for Lumbrineridae

XML Treatment for Gesaneris malaysiae

XML Treatment for Lumbrinerides acuta

XML Treatment for Nereididae

XML Treatment for Dendronereides arborifera

XML Treatment for Namalycastis cf. abiuma

XML Treatment for Namalycastis rhodochorde

XML Treatment for Neanthes glandicincta

XML Treatment for Perinereis aibuhitensis

XML Treatment for Perinereis cf. nuntia

XML Treatment for Perinereis cultrifera

XML Treatment for Perinereis rhombodonta

XML Treatment for Platynereis bicanaliculata

XML Treatment for Pseudonereis variegata

XML Treatment for Oenonidae

XML Treatment for Halla okudai

XML Treatment for Onuphidae

XML Treatment for Diopatra claparedii

XML Treatment for Diopatra neapolitana

XML Treatment for Orbiniidae

XML Treatment for Leodamas chevalieri

XML Treatment for Naineris kalkudaensis

XML Treatment for Phyllodocidae

XML Treatment for Plotohelmis capitata

XML Treatment for Rhynchonereella moebii

XML Treatment for Vanadis minuta

XML Treatment for Pilargidae

XML Treatment for Sigambra ocellata

XML Treatment for Poecilochaetus serpens

XML Treatment for Poecilochaetidae

XML Treatment for Polynoidae

XML Treatment for Drieschia pelagica

XML Treatment for Gaudichaudius cimex

XML Treatment for Olgalepidonotus kumari

XML Treatment for Sabellariidae

XML Treatment for Sabellaria jeramae

XML Treatment for Sabellidae

XML Treatment for Branchiomma nigromaculatum

XML Treatment for Caobangia abbotti

XML Treatment for Scalibregmatidae

XML Treatment for Parasclerocheilus branchiatus

XML Treatment for Sigalionidae

XML Treatment for Euthalenessa digitata

XML Treatment for Pisione oerstedii

XML Treatment for Spionidae

XML Treatment for Paraprionospio pinnata

XML Treatment for Prionospio sp.

XML Treatment for Sternaspidae

XML Treatment for Sternaspis scutata

XML Treatment for Syllidae

XML Treatment for Syllis cornuta

XML Treatment for Terebellidae

XML Treatment for Lanice socialis

XML Treatment for Tomopteridae

XML Treatment for Tomopteris (Johnstonella) aloysii sabaudiae

XML Treatment for Tomopteris (Johnstonella) dunckeri

XML Treatment for Tomopteris mariana

XML Treatment for Tomopteris nisseni

XML Treatment for Typhloscolecidae

XML Treatment for Typhloscolex muelleri

1D24DBF2-3CB1-5015-A35B-07145D421D5A 10.3897/BDJ.11.e110021.suppl1 Supplementary material 1 Checklist of species with additional information Data type Table Brief description Contains species listed in the checklist with remarks of species with type locality in Malaysia and species that were originally described outside of the Central and Western Indo-Pacific. The species that were listed in the latter were regarded as questionable species. Information on the repository and voucher materials of species were listed. On a seperate sheet is a list of species that were excluded from the previous checklist by Idris and Arshad (2013). File: oo_901762.xlsx https://binary.pensoft.net/file/901762 Raz Shauqeena Batrisyea Razmi Shah

43285B26-72C1-5C00-913A-723D8B83FC07 10.3897/BDJ.11.e110021.suppl2 Supplementary material 2 Occurrences of species Data type Table Brief description Contains the occurrences of the species with their location, depth and habitat. These data have been used to construct the bathymetry distribution map. File: oo_913194.xlsx https://binary.pensoft.net/file/913194 Raz Shauqeena Batrisyea Razmi Shah

## Figures and Tables

**Table 1.** References with records of polychaete species in Malaysian waters.

**Author (Year)**	**Number of species listed/mentioned/collected from Malaysian waters**
de Quatrefages (1866)	1
Grube (1875)	1
Horst (1911)	1
Dales (1959)	14
Chuang (1961)	2
Rullier (1969), Rullier (1970), Rullier (1976)	3
Jones (1974)	1
Nakao et al. (1989a), Nakao et al. (1989b)	10
Ong (1995)	13
Nishi (2001)	1
Paxton (2002)	1
Glasby and Hsieh (2006)	1
Idris et al. (2012)	2
Idris and Arshad (2013)	2
Idris et al. (2014)	2
Nishi et al. (2015)	1
Ibrahim et al. (2017)	1
Ibrahim et al. (2019)	1

**Table 2.** Number of species per family reported in Malaysian waters.

**Family**	**Number of species**
Nereididae de Blainville, 1818	10
Tomopteridae Grube, 1850	4
Lopadorrhynchidae Claparède, 1870	3
Phyllodocidae Örsted, 1843	3
Polynoidae Kinberg, 1856	3
Chaetopteridae Audouin and Milne Edwards, 1833	2
Eunicidae Berthold, 1827	2
Glyceridae Grube, 1850	2
Lumbrineridae Schmarda, 1861	2
Onuphidae Kinberg, 1865	2
Orbiniidae Hartman, 1942	2
Sabellidae Latreille, 1825	2
Sigalionidae Kinberg, 1856	2
Spionidae Grube, 1850	2
Amphinomidae Savigny in Lamarck, 1818	1
Aphroditidae Malmgren, 1867	1
Capitellidae Grube, 1862	1
Cirratulidae Ryckholt, 1851	1
Dorvilleidae Chamberlin, 1919	1
Eulepethidae Chamberlin, 1919	1
Iospilidae Bergström, 1914	1
Oenonidae Kinberg, 1865	1
Pilargidae de Saint-Joseph, 1899	1
Poecilochaetidae Hannerz, 1956	1
Sabellariidae Johnston, 1865	1
Scalibregmatidae Malmgren, 1867	1
Sternaspidae Carus, 1863	1
Syllidae Grube, 1850	1
Terebellidae Johnston, 1846	1
Typhloscolecidae Uljanin, 1878	1

**Table 3.** Number of families and species reported in neighbouring countries or regions.

**Country/Region**	**Families**	**Species**	**Reference**
Australia	81	1139	Beesly et al. (2000)
The China and Philippines Seas	60	1037	Salazar-Vallejo et al. (2014)
Indonesia	51	580	Pamungkas and Glasby (2019)
South China Sea	54	661	Paxton and Chou (2000)
Philippines	36	219	Paxton and Chou (2000), Salazar-Vallejo et al. (2014)
Andaman and Nicobar Islands	29	193	Rajasekaran and Fernando (2012), Muruganantham et al. (2015), Gopal et al. (2016)
Vietnam	38	191	Paxton and Chou (2000), Salazar-Vallejo et al. (2014)
Thailand	37	145	Aungtonya et al. (2002)
Singapore	28	74	Tan and Chou (1993), Chan (2009)
Malaysia	30	57	Idris and Arshad (2013); Present study

**Table 4.** List of Malaysian polychaete species collected from taxonomical and ecological literature from 1960s until recently. Repository of specimens listed, based on original literature, when available. The symbol ‘-‘ indicates either no information provided or difficulty to obtain the information as the literature is not directly accessible. The questionmark likely suggests the institution, based on the author's workplace. Acronyms of repository (in alphabetical order): **AM**: Australian Museum, Sydney; **CBM**: Natural History Museum and Institute, Chiba; **KMNH**: Kitakyushu Museum of Natural History, Kitakyushu, Fukuoka; **ICHUM**: Hokkaido University Museum, Sapporo, Hokkaido; **IEA**: Institut d’Écologie Appliquée, Université Catholique de l’Ouest, Angers; **MNHN**: Muséum National d’Histoire Naturelle, Paris, France; **NML**: Naturalis Museum, Leiden, Netherlands; **NSMT**: National Museum of Nature and Science, Tsukuba, Japan; **NTM**: Art Gallery of Northern Territory, Australia; **PMBC**: Phuket Marine Biological Centre, Phuket, Thailand; **SFRS**: Singapore Fisheries Research Station; **UM**: University of Malaya, Malaysia; **UMT**: South China Sea Repository and Reference Centre of Universiti Malaysia Terengganu, Malaysia; **USM**: Central Marine and Coastal Studies, University of Science, Malaysia; **YNU-M**: Yokohama National University Museum, Yokohama; **ZMB**: Zoologischen Museums, Berlin, Germany.

**Family**	**Species**	**Authorship, Year**	**Repository**	**Reference(s)**
Amphinomidae	*Notopygoscirratus*	Horst, 1911	NML	Salazar-Vallejo et al. (2014)
Aphroditidae	*Aphroditasondaica*	Grube, 1875	ZMB	Salazar-Vallejo et al. (2014)
Capitellidae	*Notomastuslatericeus*	Sars, 1851	-	Nakao et al. (1989b)
Chaetopteridae	*Mesochaetopterusselangora*	(Claparède, 1869)	UM & CBM	Rullier (1976), Nishi (1999)
Chaetopteridae	*Spiochaetopteruscostarum*	(Rullier, 1976)	-	Nakao et al. (1989a)
Cirratulidae	*Cirriformiatentaculata*	(Montagu, 1808)	-	Nakao et al. (1989b)
Dorvilleidae	*Protodorvilleaegena*	(Ehlers, 1913)	USM	Ong (1995)
Eulepethidae	*Grubeulepismalayensis*	Nishi, 2001	UM	Nishi (2001)
Eunicidae	Marphysacf.mossambica	Idris, Hutchings & Arshad, 2014	AM & NTM	Idris et al. (2014)
Eunicidae	*Marphysamoribidii*	(Peters, 1854)	AM & NTM	Idris et al. (2014)
Glyceridae	*Glyceraalba*	(O.F. Müller, 1776)	-	Nakao et al. (1989a)
Glyceridae	*Glyceracinnamomea*	Grube, 1874	USM	Ong (1995)
Iospilidae	*Phalacrophorusuniformis*	Reibisch, 1895	SFRS?	Dales (1959)
Lopadorrhynchidae	*Lopadorrhynchusbrevis*	Grube, 1855	SFRS?	Dales (1959)
Lopadorrhynchidae	*Maupasiacoeca*	Viguier, 1886	SFRS?	Dales (1959)
Lopadorrhynchidae	*Pelagobialongicirrata*	Greeff, 1879	SFRS?	Dales (1959)
Lumbrineridae	*Gesanerismalaysiae*	(Verrill, 1875)	MNHN & IEA	Carrera-Parra (2006)
Lumbrineridae	*Lumbrineridesacuta*	(Rullier, 1969)	-	Nakao et al. (1989b)
Nereididae	*Dendronereisarborifera*	Peters, 1854	-	Nakao et al. (1989a)
Nereididae	Namalycastiscf.abiuma	(Baird, 1863)	AM & NTM	Idris et al. (2012)
Nereididae	*Namalycastisrhodochorde*	(Grube, 1840)	AM & NTM	Idris et al. (2012)
Nereididae	*Neanthesglandicincta*	(Grube & Kröyer in Grube, 1858)	UMT, PMBC & NSMT	Ibrahim et al. (2019), Azmi et al. (2021)
Nereididae	*Perinereisaibuhitensis*	(Grube, 1872)	UMT	Ibrahim et al. (2017)
Nereididae	Perinereiscf.nuntia	Glasby, Miura, Nishi & Junardi, 2007	AM & NTM	Idris et al. (2012)
Nereididae	*Perinereiscultrifera*	(Lamarck, 1818)	-	Chuang (1961)
Nereididae	*Perinereisrhombodonta*	Wu, Sun & Yang, 1981	NTM	Glasby and Hsieh (2006)
Nereididae	*Platynereisbicanaliculata*	(Southern, 1921)	-	Nakao et al. (1989b)
Nereididae	*Pseudonereisvariegata*	(Grube, 1878)	-	Chuang (1961)
Oenonidae	*Hallaokudai*	Imajima, 1967	AM & NTM	Okuda (1933), Idris and Arshad (2013)
Onuphidae	*Diopatraclaparedii*	Grube, 1878	AM & NTM	Idris and Arshad (2013)
Onuphidae	*Diopatraneapolitana*	delle Chiaje, 1841	USM	Ong (1995)
Orbiniidae	*Leodamaschevalieri*	(Fauvel, 1902)	USM	Ong (1995)
Orbiniidae	*Naineriskalkudaensis*	(De Silva, 1965)	USM	Ong (1995)
Phyllodocidae	*Plotohelmiscapitata*	(Greeff, 1876)	SFRS?	Dales (1959)
Phyllodocidae	*Rhynchonereellamoebii*	(Apstein, 1893)	SFRS?	Dales (1959)
Phyllodocidae	*Vanadisminuta*	Treadwell, 1906	SFRS?	Dales (1959)
Pilargidae	*Sigambraocellata*	(Hartmann-Schröder, 1959)	-	Nakao et al. (1989a)
Poecilochaetidae	*Poecilochaetusserpens*	Allen, 1904	USM	Ong (1995)
Polynoidae	*Drieschiapelagica*	(de Quatrefages, 1866)	SFRS?	Dales (1959)
Polynoidae	*Gaudichaudiuscimex*	(Rullier, 1970)	MNHN	Salazar-Vallejo et al. (2014)
Polynoidae	*Olgalepidonotuskumari*	Michaelsen, 1892	MNHN	Salazar-Vallejo et al. (2014)
Sabellariidae	*Sabellariajeramae*	Nishi, Matsuo, Capa, Tomioka, Kajihara, Kupriyanova & Polgar, 2015	AM, CBM, KMNH, YNU-M & ICHUM	Nishi et al. (2015)
Sabellidae	*Branchiommanigromaculatum*	(Baird, 1865)	USM	Ong (1995)
Sabellidae	*Caobangiaabbotti*	Jones, 1974	USNM, NHM, MNHN & NML	Salazar-Vallejo et al. (2014)
Scalibregmatidae	*Parasclerocheilusbranchiatus*	Fauvel, 1928	USM	Ong (1995)
Sigalionidae	*Euthalenessadigitata*	(McIntosh, 1885)	USM	Ong (1995)
Sigalionidae	*Pisioneoerstedii*	Grube, 1857	USM	Ong (1995)
Spionidae	*Paraprionospiopinnata*	(Ehlers, 1901)	-	Nakao et al. (1989a), Nakao et al. (1989b)
Spionidae	*Prionospio* sp.		USM	Ong (1995)
Sternaspidae	*Sternaspisscutata*	(Ranzani, 1817)	-	Nakao et al. (1989a)
Syllidae	*Sylliscornuta*	Rathke, 1843	USM	Ong (1995)
Terebellidae	*Lanicesocialis*	(Willey, 1905)	USM	Ong (1995)
Tomopteridae	Tomopteris (Johnstonella) aloysii sabaudiae	Rosa, 1908	SFRS?	Dales (1959)
Tomopteridae	Tomopteris (Johnstonella) dunckeri	Rosa, 1908	SFRS?	Dales (1959)
Tomopteridae	*Tomopterismariana*	Greeff, 1885	SFRS?	Dales (1959)
Tomopteridae	*Tomopterisnisseni*	Rosa, 1908	SFRS?	Dales (1959)
Typhloscolecidae	*Typhloscolexmuelleri*	Busch, 1851	SFRS?	Dales (1959)

## References

[B9864123] Abdelnaby F. (2020). Alien Polychaete species and the first record of *Branchiommabairdi* (McIntosh, 1885) from the Suez Canal and the Mediterranean coast of Egypt. Egyptian Journal of Aquatic Biology and Fisheries.

[B9864132] Abdelnaby F. (2020). On some Nereididae (Polychaeta) with new records for the Egyptian waters. Egyptian Journal of Aquatic Biology and Fisheries.

[B9864141] Achari G. P. (1968). Studies on new or little known Polychaetes from Indian seas 1. *Trocmochaetawatsoni* (Fauvel) and *Poecilochaetusserpens* Allen. Journal of the Marine Biological Association of India.

[B9912016] Afli A., Ibin Chaabane K., Chakroun R., Jabeur C., Ramos-Esplá A. A. (2013). Specific diversity of the benthic macrofauna within the western coast of Tunis bay and the Djerba island coast (southwestern Mediterranean).. Bulletin de l'Institut National des Sciences et Technologies de la Mer.

[B9864159] Alias N. S., Hamid M. Abd, Ibrahim N. F., Bachok Z., Idris I. (2022). Polychaete Assemblages in the Sungai Terengganu Estuary (East Coast of Peninsular Malaysia): Spatial Distribution Patterns. Wetlands.

[B9864180] Al-Kandari M., Sattari Z., Hussain S., Radashevsky V. I., Zhadan A. (2019). Checklist of intertidal polychaetes (Annelida) of Kuwait, Northern part of the Arabian Gulf. Regional Studies in Marine Science.

[B9874220] Amei K., Jimi N., Kitamura M., Yokoi N., Yamaguchi A. (2020). Community structure and seasonal changes in population structure of pelagic polychaetes collected by sediment traps moored in the subarctic and subtropical western North Pacific Ocean. Zoosymposia.

[B9911998] Angsupanich S., Kuwabara R. (1999). Distribution of macrobenthic fauna in Phawong and U‐Taphao canals flowing into a lagoonal lake, Songkhla, Thailand. Lakes & Reservoirs. Research & Management.

[B9874230] Arvanitidis C. (2000). Polychaete fauna of the Aegean Sea: inventory and new information. Bulletin of Marine Science.

[B9874248] Aungtonya C., Thaipal S., Bussarawit S. (2002). A list of polychaetes (Annelida) in the reference collection database of the Phuket Marine Biological Center, Thailand. Phuket Marine Biological Center Special Publication.

[B9874239] Aungtonya C., Eibye-Jacobsen D., Kato T. (2010). The genus *Euthalenessa* (Sigalionidae: Polychaeta) from Thai and Japanese waters. Publications of the Seto Marine Biological Laboratory. Special Publication Series.

[B9874267] Azmi S. S., Ibrahim Y. S., Angsupanich S., Sumpuntarat P., Sato M. (2021). Epitokous metamorphosis, reproductive swimming, and early development of the estuarine polychaete, *Neanthesglandicincta* Southern, 1921 (Annelida, Nereididae) on the east coast of the Malay Peninsula. Zookeys.

[B9874277] Baert J. L., Slomianny M. C. (1987). Heterosynthetic origin of the major yolk protein, vitellin, in a nereid, *Perinereiscultrifera* (polychaete annelid). Comparative Biochemistry and Physiology Part B: Comparative Biochemistry.

[B9874312] Barnich R., Fiege D., Sun R. (2004). Polychaeta (Annelida) of Hainan Island, South China Sea Part III. Aphroditoidea. Species Diversity.

[B9874323] Batistić M., Kršinić F., Jasprica N., Carić M., Viličić D., Lučić D. (2004). Gelatinous invertebrate zooplankton of the South Adriatic: species composition and vertical distribution. Journal of Plankton Research.

[B10035319] Baustian M. M., Bargu S., Morrison W., Sexton C., Rabalais N. N. (2018). The polychaete, *Paraprionospiopinnata*, is a likely vector of domoic acid to the benthic food web in the northern Gulf of Mexico. Harmful Аlgae.

[B9874334] Beesly P. L., Ross G. J.B., Glasby C. J. (2000). Polychaete & Allies: The Southern synthesis. Fauna of Australia. Vol. 4A Polychaeta, Myzostomida, Pogonophora, Echiura, Sipuncula.

[B9874344] Bilbao M., Palma S., Rozbaczylo N. (2008). First records of pelagic polychaetes in southern Chile (Boca del Guafo-Elefantes Channel). Latin American Journal of Aquatic Research.

[B9911904] Blackstock J., Barnes M. (1982). The Loch Eil project: biochemical composition of the polychaete, *Glyceraalba* (Müller), from Loch Eil. Journal of Experimental Marine Biology and Ecology.

[B9874353] Bleeker J., van der Spoel S. (1992). Catalogue of the Polychaeta collected by the Siboga Expedition and type specimens of Polychaeta in the Zoological Museum of Amsterdam. Bulletin Zoologisch Museum.

[B9874362] Böggemann M. (2002). Revision of the Glyceridae Grube 1850 (Annelida: Polychaeta). Abhandlungen der Senckenbergischen Naturforschenden Gesellschaft.

[B9874371] Böggemann M., Eibye-Jacobsen D. (2002). The Glyceridae and Goniadidae (Annelida: Polychaeta) of the BIOSHELF Project, Andaman Sea, Thailand.. Phuket Marine Biological Center Special Publication.

[B9874388] Buchman N., Cuddington K., Lambrinos J., Cuddington K., Byers J. E., Wilson W. G., Hastings A. (2007). Ecosystem engineers: plants to protists.

[B9874409] Buyck B. (1999). Taxonomists are an endangered species in Europe. Nature.

[B9874418] Cantone G. (1982). Researches on the coast of Somalia. Polychaetous annelids of Mogadiscio, Gesira, Bender Mtoni and Sar Uanle. Monitore Zoologico Italiano.. Supplemento.

[B9874427] Carrera-Parra L. F. (2006). Phylogenetic analysis of Lumbrineridae Schmarda, 1861 (Annelida: Polychaeta). Zootaxa.

[B9874436] Chan W. M.F. (2009). New nereidid records (Annelida: Polychaeta) from mangroves and sediment flats of Singapore. Raffles Bulletin of Zoology.

[B9874445] Che Soh N. A., Rapi H. S., Mohd Azam N. S., Santhanam R. K., Assaw S., Haron M. N., Ismail W. I.W. (2020). Acute wound healing potential of marine worm, *Diopatraclaparedii* Grube, 1878 aqueous extract on sprague dawley rats. Evidence-Based Complementary and Alternative Medicine.

[B9874472] Chuang S. H. (1961). On Malayan shores/ S.H. Chuang.

[B9911952] Çinar M. E., Ergen Z., Ozturk B., Kirkim F. (1998). Seasonal analysis of zoobenthos associated with a *Zosteramarina* L. bed in Gulbahce Bay Aegean Sea, Turkey. Marine Ecology.

[B9874482] Çinar M. E., Dağli E., Şahin G. K. (2014). Checklist of Annelida from the coasts of Turkey. Turkish Journal of Zoology.

[B9874491] (CBD) Convention on Biological Diversity Biodiversity Facts. Secretariat of the Convention on Biological Diversity (SCBD). https://www.cbd.int/countries/profile/?country=my.

[B9874520] Dağli E., Ergen Z., Cinar M. E. (2005). One year observation on the population structure of *Diopatraneapolitana* Delle Chiaje (Polychaeta: Onuphidae) in Izmir Bay (Aegean Sea, eastern Mediterranean). Marine Ecology.

[B9874554] Dales R. P. (1955). The pelagic polychaetes of Monterey Bay, California.. Annuals and Magazine of Natural History.

[B9876993] Dales R. P. (1957). Pelagic polychaetes from the Bay of Biscay. Annals and Magazine of Natural History.

[B9874545] Dales R. P. (1957). Pelagic Polychaetes of the Pacific Ocean. UC San Diego: Bulletin of the Scripps Institution of Oceanography.

[B9874563] Dales R. P. (1959). Pelagic polychaetes from the Malacca Straits and south China Sea.. Journal of Natural History.

[B9874572] Dana-Report (1934). The Carlsberg Foundation’s Oceanographic Expedition round the World 1928–30 and previous ‘Dana’-expeditions under the leadership of the late Professor Johannes Schmidt. Vol. 1. No. 1. Introduction to the reports from the Carlsberg Foundation’s Oceanographic Expedition round the World 1928–30.. CA Reitzels Forlag, Copenhagen.

[B9913617] Dauvin J. C., Thiebaut E. (1994). Is *Oweniafusiformis* Delle Chiaje a cosmopolitan species?. Mémoires du Muséum national D’histoire naturelle.

[B9874581] Day J. H. (1967). A monograph on the Polychaeta of Southern Africa. Part 1. Errantia. British Museum (Natural History), London.

[B10080450] de Quatrefages A (1866). Histoire naturelle des Annelés marins et d'eau douce. Annélides et Géphyriens..

[B10455496] De Silva P. H. (1965). New species and records of Polychaeta from Ceylon. Proceedings of the Zoological Society of London.

[B9876145] Elayaraja S., Murugesan P., Vijayalakshmi S., Balasubramanian T. (2010). Antibacterial and antifungal activities of polychaete *Perinereiscultrifera*. Indian Journal of Geo-Marine Sciences.

[B9912007] Esquete P., Ramos E., Riera R. (2016). New data on the Tanaidacea (Crustacea: Peracarida) from the Canary Islands, with a description of a new species of *Apseudopsis*. Zootaxa.

[B10089989] Eydoux F., Souleyet L. F.A. (1842). Voyage Autour du Monde, Exécuté Pendant les Années 1836 et 1837 sur la Corvette La Bonite, Commandée par M. Vaillant.

[B10080415] Fadhullah W., Syakir M. I., Ahmad M. I., Ismail M., Riffat S. (2016). Renewable energy and sustainable technologies for building and environmental applications.

[B10455486] Fauvel P. (1902). Annélides Polychètes de la Casamance rapportées par M. Aug. Chevalier.. Bulletin de la Société Linnéenne de Normandie.

[B9876182] Fauvel P. (1923). Faune de France 5: Polychètes errantes.

[B9876190] Fauvel P. (1932). Annelida
Polychaeta of the Indian Museum, Calcutta. Memoirs of the Indian Museum.

[B10080402] Fauvel P., Sewell R. S. (1953). The fauna of India, including Pakistan,Ceylen, Burma and Malaya.

[B9876242] Fernández-Álamo M. A. (1991). Holoplanktonic polychaetes from the Gulf of the Gulf of California: August–September 1977.

[B9876229] Fernández-Álamo M. A., Thuesen E., Boltovskoy D. (1999). South Atlantic Zooplankton..

[B9876250] Fernández-Álamo M. A., Sanvicente-Añorve L. (2005). Holoplanktonic polychaetes from the Gulf of Tehantepec, Mexico. Cahiers de Biologie Marine.

[B9913432] Fong P. P. (1993). Lunar control of epitokal swarming in the polychaete *Platynereisbicanaliculata* (Baird) from central California. Bulletin of Marine Science.

[B9876259] Freeman D. B. (2003). The Straits of Malacca: Gateway or Gauntlet?.

[B9913441] Fukao R. (1996). Occurrence of epitokes of *Platynereisbicanaliculata* (Baird) (Annelida: Polychaeta) in Koajiro Bay, Miura peninsula, Central Japan. Publications of the Seto Marine Biological Laboratory.

[B9876267] Gambi M. C., Giangrande A. (1986). Distribution of soft-bottom polychaetes in two coastal areas of the Tyrrhenian Sea (Italy): structural analysis. Estuarine, Coastal and Shelf Science.

[B9876276] Gholizadeh M., Yahya K., Talib A., Ahmad O. (2012). Effects of environmental factors on polychaete assemblage in Penang National Park, Malaysia. Proceedings of World Academy of Science, Engineering and Technology.

[B9911913] Giangrande A., Fraschetti S. (1993). Life cycle, growth and secondary production in a brackish‐water population of the polychaete *Notomastuslatericeus* (Capitellidae) in the Mediterranean Sea. Marine Ecology.

[B9876303] Glasby C. J., Hutchings P. A., Fauchald K., Paxton H., Rouse G. W., Russell C. W., Wilson S., Beesly P. L., Ross G. J.B., Glasby C. J. (2000). Polyhchaete & allies: The Southern Synthesis. Fauna of Australia.

[B9876294] Glasby C. J., Hsieh H. L. (2006). New species and new records of the *Perinereisnuntia* species group (Nereididae: Polychaeta) from Taiwan and other Indo-West Pacific shores. Zoological Studies-Taipei.

[B9876319] Glasby C. J., Lee Y. L., Hsueh P. W. (2016). Marine Annelida (excluding clitellates and siboglinids) from the South China Sea. Raffles Bulletin of Zoology.

[B10515135] Glasby C. J., Al Hakim I. (2017). History of collection and discovery of polychaetes (Annelida), including a bibliography, from the Indo-Malay-Philippines Archipelago and surrounding seas. Raffles Bulletin of Zoology.

[B9876285] Glasby C. J., Erséus C., Martin P. (2021). Annelids in extreme aquatic environments: diversity, adaptations, and evolution. Diversity.

[B9876354] Gopal A. K.U., Parameswaran A. J.U.V., Vijayan A. K. (2016). *Armandiasampadae*, a new species of polychaete (Opheliidae) from Andaman Sea, Northern Indian Ocean. Journal of the Marine Biological Association of the United Kingdom.

[B10455544] Grube A. E. (1857). Annulata Orstediana. Enumeratio Annulatorum, quac in itinere per Indiam occidentalem et Americam centralem annis 1845-1848 suscepto legit cl. A. S. Orsted, adjectis speciebus nonnullis a cl. H. Kroyero in itinere ad Americam meridionalem collectis. Videnskabelige Meddelelser fra Dansk Naturhistorisk Forening i Köbenhavn.

[B9876372] Grube A. E. (1875). Bemerkungen über die Familie der Aphroditen. (Gruppe Hermionea und Sigalionina). Jahresbericht der Schlesischen Gesellschaft für Vaterländische Cultur.

[B9876381] Grube A. E. (1878). Annulata Semperiana. Beiträge zur Kenntniss der Annelidenfauna der Philippinen nach den von Herrn Prof. Semper mitgebrachten Sammlungen. Mémoires de l’Académie impériale des sciences de St Petersbourg, Serie 7.

[B9876390] Guglielmo R., Gambi M. C., Granata A., Guglielmo L., Minutoli R. (2014). Composition, abundance, and distribution of holoplanktonic polychaetes within the Strait of Magellan (southern America) in austral summer. Polar Biology.

[B9876400] Hamzah S. R., Anuar S. T., Khalik W. M.A.W.M., Kolandhasamy P., Ibrahim Y. S. (2021). Ingestion of microplastics by the estuarine polychaete, *Namalycastis* sp. in the Setiu Wetlands, Malaysia. Marine pollution bulletin.

[B9911895] Harkantra S. N., Parulekar A. H. (1985). Community structure of sand-dwelling macrofauna of an estuarine beach in Goa, India.. Marine Ecology-Progress Series.

[B9876419] Hernández-Alcántara P., Tovar-Hernández M. A., Solís-Weiss V. (2008). Polychaetes (Annelida: Polychaeta) described for the Mexican Pacific: an historical review and an updated checklist. Latin American Journal of Aquatic esearch.

[B9876410] Hernández-Alcántara P., Solís-Weiss V. (2013). Biodiversity and distribution of the *Scolecida* (Annelida: Polychaeta) on the continental shelf of the Gulf of California, Mexican Pacific. Cahiers de Biologie Marine.

[B9876428] Herwerden V. L. (1989). Collection of mussel worms *Pseudonereisvariegata* for bait-a legislative anachronism. South African Journal of Marine Science.

[B9876456] Horst R. (1911). On the genus *Notopygos*, with some new species from the Malay-Archipelago collected by the Siboga-Expedition. Notes from the Leyden Museum.

[B10081400] Horst R. (1924). Polychaeta
errantia of the Siboga-Expedition. Part Ⅲ. Nereidae and Hesionidae. Siboga-Expedition Leyden.

[B9876446] Hussain N. S., Harun N. A., Mohd Radzi M. N.F., Idris I., Wan Ismail W. I. (2018). Biosynthesis of silver nanoparticles from marine polychaete *Diopatraclaparedii* Grube, 1878. Jurnal Teknologi.

[B10081391] Hutchings P., Reid A., Wilson R. (1991). *Perinereis* (Polychaeta: Nereididae) from Australia, with redescriptions of six additional species. Records of the Australian Museum.

[B9876465] Hutchings P., Kupriyanova E. (2018). Cosmopolitan polychaetes-fact or fiction? Personal and historical perspectives. Invertebrates Systematics.

[B10081382] Hylleberg J., Nateewathana A., Bussarawit S. (1986). Polychaetes of Thailand. Nereidae (Part 1): *Perinereis* and *Psedonereis* with notes on species of commercial value. Phuket Marine Biology Center Research Bulletin.

[B9876474] Ibrahim N. F., Ibrahim Y. S., Sato M. (2019). New record of an estuarine polychaete, *Neanthesglandicincta* (Annelida, Nereididae) on the eastern coast of Peninsular Malaysia. ZooKeys.

[B10081275] Ibrahim Y. S., Ibrahim N. F., Idris I., Faridah M., Ibrahim Y. S., Baharuddin N., Abd Rahman Azmi A. A., Borkhanuddin M. H. (2017). Invertebrates of Setiu Wetlands.

[B9876529] Idris I., Glasby C., Arshad A., Mohamed-Zin W. W.M.K (2012). Nereididae (Annelida: Polychaeta) used as baitworms in Peninsular Malaysia. UMT Postgraduate Conference.

[B9876483] Idris I., Arshad A. (2013). Checklist of polychaetous annelids in Malaysia with redescription of two commercially exploited species. Asian Journal of Animal and Veterinary Advances.

[B9876545] Idris I., Hutchings P., Arshad A. (2014). Description of a new species of *Marphysa* Quatrefages, 1865 (Polychaeta: Eunicidae) from the west coast of Peninsular Malaysia and comparisons with species from *Marphysa* Group A from the Indo-West Pacific and Indian Ocean. Memoirs of Museum Victoria.

[B9876554] Idris I., Mohd-Salleh N. A., Ahmad Fadzil N. D.N., Chuan O. M., Martin M. B., Nurulnadia M. Y., Azmi W. A. (2022). Bidong Island: Natural History and Resources.

[B9966454] Imajima M., Hartman O. (1964). The polychaetous annelids of Japan. Part II. Allan Hancock Foundation Publications, Occasional Paper.

[B10080385] Ismail Siti Zubaidah, Mohd Sani Mohd Azizuddin (2010). The Straits of Malacca: Regional powers vis-a-vis littoral states in strategic and security issues and interests..

[B9876583] Jiménez-Cueto S., Suárez-Morales E. (2008). An account of *Alciopina*, *Torrea*, and *Rhynconereella* (Polychaeta: Alciopidae) of the western Caribbean Sea. Belgian Journal of Zoology.

[B9876592] Jones M. L. (1974). On the Caobangiidae, a new family of the Polychaeta, with a redescription of *Caobangiabilleti* Giard.

[B9876613] Junardi, A Ridwan, Yuwono E., Anggraeni T. (2014). The maturity of nypa palm worm *Namalycastisrhodochorde* (Polychaeta: Nereididae. AIP Conference Proceedings.

[B9876600] Junardi, Kustiati, Candramila W. (2020). KOBI-2019 (EPiC Series in Biological Sciences).

[B9912044] Kinner P., Maurer D. (1978). Polychaetous annelids of the Delaware Bay region. Fishery Bulletin.

[B9876623] Knowlton N. (1993). Sibling species in the sea. Annual Review of Ecology, Evolution, and Systematics.

[B9876632] Knowlton N. (2000). Molecular genetic analyses of species boundaries in the sea. Hydrobiologia.

[B9876641] Kobayashi G., Mukai R., Itoh H. (2020). New record of *Hallaokudai* Imajima, 1967 (Annelida, Eunicida, Oenonidae) from Fukue Island in the Goto Islands, Japan. Check List.

[B9876650] Kolbasova G., Neretina T. (2021). A new species of *Pelagobia* (Lopadorrhynchidae, Annelida), with some notes on literature records of *Pelagobialongicirrata* Greeff, 1879. Zootaxa.

[B10034294] Lee Y. L., Glasby C. J. (2015). A new cryptic species of *Neanthes* (Annelida: Phyllodocida: Nereididae) from Singapore confused with *Neanthesglandicincta* Southern, 1921 and Ceratonereis (Composetia) burmensis (Monro, 1937). Raffles Bulletin of Zoology.

[B9876659] Lewis J. A., Dunstan I. C., Forsyth J. R. (1981). Biological survey of marine communities around Triangular Island (Shoalwater Bay, Queensland). Materials Research Labs Ascot Vale (Australia).

[B9913461] Lim H. S., Hong J. S. (1997). Ecology of the macrozoobenthos in Chinhae Bay, Korea 2. Distribution pattern of the major dominant species. Korean Journal of Fisheries and Aquatic Sciences.

[B9876686] Londoño-Mesa M., Polanía J., Vélez I. (2002). Polychaetes of the mangrove-fouling community at the Colombian Archipelago of San Andrés and Old Providence, Western Caribbean. Wetlands Ecology and Management.

[B9911886] Macnae W., Kalk M. (1962). The Fauna and Flora of Sand Flats at Inhaca Island, Mozambique. The Journal of Animal Ecology.

[B9876704] Maltagliati F., Camilli L., Lardicci C., Castelli A. (2001). Evidence for morphological and genetic divergence in *Perinereiscultrifera* (Polychaeta: Nereididae) from two habitat types at Elba Island. Journal of the Marine Biological Association of the United Kingdom.

[B9876713] Martin D., Britayev T. A. (1998). Symbiotic polychaetes: review of known species. Oceanography and Marine Biology: An Annual Review.

[B9876722] Mohammad M. B. (1980). Polychaete annelids from Kuwaitian islands, Arabian Gulf, with descriptions of four new species. Zoological Journal of the Linnean Society.

[B10034303] Monro C. C.A. (1937). On two new polychaetes from Indian Ocean. Annals and Magazine of Natural History Series.

[B9876740] Muruganantham M., Mohan P. M., Karunakumari R., Ubare V. V. (2015). First report of Nereis (Neanthes) virens (Sars) an epitoky polychaete worm from Middle Strait, Baratang, Andaman Island, India. Journal of Research in Biology.

[B10080344] Murugesan P., Balasubramanian T., Rastogi R. P., Phulwaria M., Gupta D. K. (2021). Mangroves: Ecology, Biodiversity and Management.

[B9876767] Nakao S., Shazili N. A.M., Salleh H. U. (1989). Benthic communities in the areas under and around the fish-culture rafts at the Kuala Terengganu River Estuary, Malaysia. Bulletin of the Faculty of Fisheries Hokkaido University.

[B9876758] Nakao S., Nomura H., Kamal M., Satar B. A. (1989). Macrobenthos and sedimentary environments in a Malaysian intertidal mudflat of the Cockle Bed. Bulletin of the Faculty of Fisheries Hokkaido University.

[B9876776] Neave M. J., Glasby C. J., McGuinness K. A., Parry D. L., Streten-Joyce C., Gibb K. S. (2013). The diversity and abundance of polychaetes (Annelida) are altered in sediments impacted by alumina refinery discharge in the Northern Territory, Australia. Marine Environmental Research.

[B9876787] Neumann-Leitão S., Santánna E. M.E., Gusmão L. M.D.O., Do Nascimento-Vieira D. A., Paranaguá M. N., Schwamborn R. (2008). Diversity and distribution of the mesozooplankton in the tropical Southwestern Atlantic. Journal of Plankton Research.

[B9876798] Ng P. K.L., Sasekumar A. (1993). A new species of *Polyonyx* Stimpson, 1858, of the *P.sinensis* group (Crustacea: Decapoda: Anomura: Porcellanidae) commensal with a chaetopterid worm from Peninsular Malaysia. Zoologische Mededelingen.

[B9911962] Nishi E., Arai Y. (1996). Chaetopterid polychaetes from Okinawa Island, Japan, with notes on the feeding behaviour of *Spiochaetopteruscostarumcostarum*. Publications of the Seto Marine Biological Laboratory.

[B9876807] Nishi E. (1999). Redescription of *Mesochaetopterusselangolus* (Polychaeta: Chaetopteridae), based on type specimens and recently collected material from Morib Beach, Malaysia. Pacific Science.

[B9876816] Nishi E. (2001). A new species of scaleworm, *Grubeulepismalayensis* (Annelida: Polychaeta: Eulepethidae), from Morib Beach, Malaysia, living in chaetopterid tubes. Species Diversity.

[B9876825] Nishi E., Matsuo K., Capa M., Tomioka S., Kajihara H., Kupriyanova E. K., Polgar G. (2015). *Sabellariajeramae*, a new species (Annelida: Polychaeta: Sabellariidae) from the shallow waters of Malaysia, with a note on the ecological traits of reefs. Zootaxa.

[B9876837] Okuda S. (1933). Some polychaete annelids used as bait in the inland sea. Annotationes Zoologicae Japonenses.

[B9876846] Olive P. J.W. (1994). Polychaeta as a world resource: A review of patterns of exploitation as sea angling baits and the potential for aquaculture-based production. Memorial Museum National History Natural.

[B9876855] Olive P. J.W. (1999). Polychaete aquaculture and polychaete science: A mutual synergism. Hydrobiologia.

[B9876864] Ong B. (1995). Polychaetes of Telok Aling, Penang, Malaysia. Raffles Bulletin of Zoology.

[B9876873] Orensanz J. M., Ramírez F. (1973). Taxonomía y distribución de los poliquetos pelágicos del Atlántico Sudoccidental. Boletín del Instituto de Biología Marina, Mar del Plata.

[B9876882] Pamungkas J., Glasby C. J. (2019). Status of polychaete (Annelida) taxonomy in Indonesia, including a checklist of Indonesian species. Raffles Bulletin of Zoology.

[B9876891] Pamungkas J., Glasby C. J., Read G. B., Wilson S. P., Costello M. J. (2019). Progress and perspectives in the discovery of polychaete worms (Annelida) of the world. Helgoland Marine Research.

[B9876901] Pati S. K., Swain D., Sahu K. C., Sharma R. M. (2015). Diversity and distribution of polychaetes (Annelida: Polychaeta) along Maharashtra coast, India. Aquatic Ecosystem.

[B9876937] Paxton H., Chou L. M. (2000). Polychaetous annelids from the South China Sea. Raffles Bulletin of Zoology.

[B9876910] Paxton H. (2002). *Diopatra* Audouin and Milne Edwards (Polychaeta: Onuphidae) from Thailand. Phuket Marine Biological Center Special Publication.

[B9876919] Paxton H. (2009). Phylogeny of Eunicida (Annelida) based on morphology of jaws. Zoosymposia.

[B9876928] Paxton H., Arias A. (2017). Unveiling a surprising diversity of the genus *Diopatra* Audouin & Milne Edwards, 1833 (Annelida: Onuphidae) in the Macaronesian region (eastern North Atlantic) with the description of four new species. Zootaxa.

[B9876946] Pei A. U.E., Huai P. C., Masimen M. A.A., Ismail W. I.W., Idris I., Harun N. A. (2020). Biosynthesis of gold nanoparticles (AuNPs) by marine baitworm *Marphysamoribidii* Idris, Hutchings, and Arshad, 2014 (Annelida: Polychaeta) and its antibacterial activity. Advances in Natural Sciences: Nanoscience and Nanotechnology.

[B9876957] Pettibone M. H. (1963). Marine polychaete worms of the New England region. I. Aphroditidae through Trochochaetidae. Bulletin of the United States National Museum.

[B9913484] Pettibone M. H. (1966). Revision of the Pilargidae (Annelida: Polychaeta), including descriptions of new species, and redescriptions of the pelagic *Podarmusploa* Chamberlain (Polynoidae). Proceedings of the United States National Museum.

[B9876966] Pettibone M. H. (1986). Review of the *Iphioninae* (Polychaeta: Polynoidae) and revision of *Ilphionecimex* Quatrefages, *Gattyanadeludens* Fauvel, and *Harmothoeiphionelloides* Johnson (Harmothoinae). Smithsonian Contributionsto Zoology.

[B9876975] Pettibone M. H. (1995). New genera for two polychaetes of Lepidonotinae. Proceedings of the Biological Society of Washington.

[B9876984] Phan T. (2015). Polychaetes species composition in Nha Trang bay. Collection of Marine Research Works.

[B9911943] Pienkowski M. W. (1983). Surface activity of some intertidal invertebrates in relation to temperature and the foraging behavior of their shorebird predators. Marine ecology progress series. Oldendorf.

[B9912053] Pillay D., Perissinotto R. (2008). The benthic macrofauna of the St. Lucia Estuary during the 2005 drought year. Estuarine, Coastal and Shelf Science.

[B9877002] Pinca S., Dallot S. (1995). Meso- and macrozooplankton composition patterns related to hydrodynamic structures in the Ligurian Sea (Trophos-2 experiment, April-June 1986. Marine Ecology Progress Series.

[B9877011] Pleijel F., Dales R. P., Kermack D. M., Barnes R. S.K. (1991). Synopses of the British Fauna (New Series.

[B9877024] Pocklington P., Wells P. G. (1992). Polychaetes key taxa for marine environmental quality monitoring. Marine Pollution Bulletin.

[B9877033] Polgar G., Nishi E., Idris I., Glasby C. J. (2015). Tropical polychaete community and reef dynamics: insights from a Malayan *Sabellaria* (Annelida: Sabellariidae) reef. Raffles Bulletin of Zoology.

[B9877054] Rajasekaran R., Fernando O. J., Venkataraman K., Raghunathan C., Sivaperuman C. (2012). Ecology of Faunal Communities on the Andaman and Nicobar Islands.

[B10455508] Rathke H. (1843). Beiträge zur Fauna Norwegens. Nova Acta Academiae Caesareae Leopoldino-Carolinae Naturae Curiosorum. Breslau & Bonn.

[B9877075] Read G., Fauchald K. World Polychaeta Database. https://www.marinespecies.org/polychaeta.

[B9877083] Reish D. J. (1984). Marine ecotoxicological tests with polychaetous annelids. Ecotoxicological testing for the marine environment.

[B9877092] Rezai H., Yusoff F. M., Arshad A., Kawamura A., Shariff M., Ibrahim H. M., Tan S. G., Tai S. Y. (2002). Tropical Marine Environment: Charting Strategies for the Millennium.

[B9913493] Rivera C. G., Rivera M. Y.R. (2008). Checklist of polychaetes (Annelida: Polychaeta) from El Salvador, eastern Pacific. Check List.

[B9913502] Roe P. (1975). Aspects of life history and of territorial behavior in young individuals of *Platynereisbicanaliculata* and *Nereisvexillosa* (Annelida, Polychaeta).. Pacific Science.

[B10455526] Rosa D. (1908). Raccolte planctonische fatte dalla R. Nave Liguria nel vaggio di circonnavigazione del 1903-1905. Sotto il Commando di S.A.R. Luigi di Savoia, Duca degli Abruzzi. Publd. Inst. Firenze.

[B9877117] Rosli N. S., Yahya N., Idris I., Bachok Z. (2018). Polychaetous annelid community structure in relation to soft bottom sediment characteristics in continental shelf of the southern South China Sea. Journal of Sustainability Science and Management.

[B9877126] Rouabah A., Scaps P. (2003). Life cycle and population dynamics of the polychaete *Perinereiscultrifera* from the Algerian Mediterranean Coast. Marine Ecology.

[B9877135] Rouhi A., Sif J., Ferssiwi A., Gillet P., Deutch B. (2008). Reproduction and population dynamics of *Perinereiscultrifera* (Polychaeta: Nereididae) of the Atlantic coast, El Jadida, Morocco. Cahiers de Biologie Marine.

[B9877145] Rouse G. W., Yule C. M., Sen Y. S. (2004). Freshwater Invertebrates of the Malaysian region.

[B9913511] Roy M., Nandi N. C. (2012). Distribution pattern of macrozoobenthos in relation to salinity of Hugli-Matla estuaries in India. Wetlands.

[B9877166] Rozbaczylo N. (1985). Los anélidos poliquetos de Chile. Índice sinonímico y distribución geográfica de especies. Ediciones Pontificia Universidad Católica de Chile. Serie Monografías Biológicas.

[B9877175] Rullier F. (1969). Une nouvelle espèce d'Annélide Polychète *Lumbriconereismalaysiae*. Bulletin de la Société Zoologique de France.

[B9877184] Rullier F. (1970). *Lepidonotuskumari*, une nouvelle espèce d'Aphroditidae (Annélide Polychète) de Malaisie. Bulletin de la Société Zoologique de France.

[B9877193] Rullier F. (1976). Description d'une nouvelle espece de Chaetopteridae
*Sasekumariaselangola* (Annelides Polychetes) de Malaisie. Bulletin de la Société Zoologique de France.

[B10081373] Russell E. (1962). Some nereid polychaetes from Queensland. University of Queensland Papers, Department of Zoology.

[B9877211] Saito H., Kawai K., Umino T., Imabayashi H. (2014). Fishing bait worm supplies in Japan in relation to their physiological traits. Memoirs of Museum Victoria.

[B9877229] Salazar-Vallejo S. I., Carrera-Parra L. F., Muir A. I., de Léon-González J. A., Piotrowski C., Sato M. (2014). Polychaete species (Annelida) described from the Philippine and China Seas. Zootaxa.

[B9877220] Salazar-Vallejo S. I. (2020). Practical methods for the morphological recognition and definition of genera, with a comment on polychaetes (Annelida). Biología y Sociedad.

[B9877241] Salazar-Vallejo S. I., González-Vallejo N. E. (2020). Revisiones taxonómicas, ciencia de frontera y programas nacionales. Biología y Sociedad.

[B9877425] San Martin G., López E. (2000). Three new species of *Syllis* (Syllidae: Polychaeta) from Iberian coasts. Cahiers de Biologie Marine.

[B9877434] Sarkar J., Talukdar S. (2003). Marine invertebrates of Digha coast and some recommendations on their conservation. Records of the Zoological Survey of India.

[B9913555] Sarma D. V., Rao D. (1982). Abundance and distribution of *Dendronereisarborifera* Peters, 1854 (Nereidae: Polychaeta) in the Vasishta Godavari Estuary. Indian Journal of Marine Sciences.

[B9877443] Sasekumar A. (1974). Distribution of macrofauna on a Malayan mangrove shore. The Journal of Animal Ecology.

[B9877452] Sendall K., Salazar-Vallejo S. I. (2013). Revision of *Sternaspis* Otto, 1821 (Polychaeta, Sternaspidae). ZooKeys.

[B9877461] Shi G. W., Ghaffar M. A., Ali M. M., Cob Z. C. (2014). The Polychaeta (Annelida) communities of the Merambong and Tanjung Adang Shoals, Malaysia, and its relationship with the environmental variables. Malayan Nature Journal.

[B9877470] Siciński J. (1988). Pelagic Polychaeta in the Scotia Front west of Elephant Island (BIOMASS III, October-November 1986). Polish Polar Research.

[B9877479] Siddiqui N., Mustaquim J. (1988). Four new records of nereid worms (Polychaeta: Annelida) from Karachi. Pakistan Journal of Zoology.

[B9877488] Simon C. A., Niekerk H. H., Burghardt I., ten Hove H. A., Kupriyanova E. K. (2019). Not out of Africa: *Spirobranchuskraussii* (Baird, 1865) is not a global fouling and invasive serpulid of Indo-Pacific origin. Aquatic Invasions.

[B9877498] Solis-Weiss V., Bertrand Y., Helleouet M. N., Pleijel F. (2004). Types of polychaetous annelids at the Muséum national d'Histoire naturelle, Paris. Zoosystema.

[B9877507] Soota T. D., Misra A., Chakraborty R. K. (1981). Polychaete fauna of Gujarat coast. Records of the Zoological Survey of India.

[B10034285] Southern Rowland (1921). Polychaeta of the Chilka Lake and also of fresh and brackish waters in other parts of India. Memoirs of the Indian Museum.

[B9877516] Srikrishnadhas B., Ramamoorthi K., Balasubrahmanyan K. (1987). Polychaetes of Porto Novo waters. Journal of the Marine Biological Association of India.

[B9877525] Tan L. T., Chou L. M. (1993). Checklist of polychaete species from Singapore waters (Annelida. Raffles Bulletin of Zoology.

[B9877534] Tebble N. (1962). The distribution of pelagic polychaetes across the North Pacific Ocean. Bulletin of the Natural History Museum.

[B9877543] Tovar-Faro B., Leocádio M., Paiva P. C. (2013). Distribution of Iospilidae (Annelida) along the eastern Brazilian coast (from Bahia to Rio de Janeiro). Latin American Journal of Aquatic Research.

[B10035370] Townsend M., Worsfold T. M., Smith P. R., Martina L. J., McNeill C. L., Kendall M. A. (2006). Occurrence of *Sternaspisscutata* (Polychaeta: Sternaspidae) in the English Channel. Cahiers de Biologie Marine.

[B9877552] Treadwell A. L. (1943). Polychaete annelids. Scientific Results of Cruise VII of the Carnegie during 1928-1929 under the command of Captain J.P. Ault. Biology IV. Carnegie Institution of Washington Publication.

[B9877561] Uschakov P. V., Wu B. (1959). The polychaetous annelids of the families Phyllodocidae and Aphroditidaefrom the Yellow Sea. Archives of the Chinese Institute of Oceanology.

[B9877570] Uschakov P. V., Wu B. (1965). The Polychaeta
Errantia of the Yellow Sea. Explorations of the Fauna of the Seas.

[B9877579] Uschakov P. V., Wu B. (1979). Polychaeta
Errantia of the Yellow Sea.

[B9877587] Wallace A. R. (1869). The Malay Archipelago: The Land of the Orang Utan and the Bird of Paradise. A narrative of travel, with studies of man and nature.

[B9877595] Wehe T., Fiege D. (2002). Annotated checklist of the polychaete species of the seas surrounding the Arabian Peninsula: Red Sea, Gulf of Aden, Arabian Sea, Gulf of Oman, Arabian Gulf. Fauna of Arabia.

[B9877604] Wesenberg-Lund E. (1939). Polychètes et Gephyriens de Tunisie. Bulletin de la Station Océanographique de Salammbô.

[B9913608] Williams S. J. (1984). The status of *Terebellidesstroemii* (Polychaeta, Trichobranchidae) as a cosmopolitan species on a worldwide morphological survey, including description of a new species.

[B9877622] Wu B., Sun R., Chen M. (1980). Zoogeographical studies on Polychaeta from the Xisha Islands and its adjacent waters. Acta Oceanologica Sinica.

[B9874303] Wu B, Ding Z, Huang F (1998). Preliminary study on pisionids (Annelida: Polychaeta
Pisionidae) from Hainan Island coastal waters, South China Sea. Chinese Journal of Oceanology and Limnology.

[B10455566] Wu B. L., Sun R., Yang D. (1981). The Nereidae (Polychaetous Annelids) of the Chinese Coast. Institute of Oceanology, Academia Sinica. Qingdao (Tsingtao), China.

[B10081364] Wu S. K. (1967). The nereid worms of Taiwan. Bulletin of the Institute of Zoology, Academia Sinica.

[B9911990] Yang D., Sun R. (1988). Polychaetous annelids commonly seen from the Chinese waters.

